# Combination of quantitative MRI and laboratory markers for the detection and staging of metabolic dysfunction-associated steatotic liver disease

**DOI:** 10.1007/s00330-026-12451-5

**Published:** 2026-03-28

**Authors:** Nienke P. M. Wassenaar, Koen C. van Son, Bas Voermans, Kirsi M. A. van Eekhout, Marian A. Troelstra, Stan Driessen, Anne Line Mak, Julia J. Witjes, Anne-Marieke van Dijk, Veera Houttu, Diona Zwirs, Elizabeth Shumbayawonda, Max Nieuwdorp, Michail Doukas, Joanne Verheij, Aart J. Nederveen, Oliver J. Gurney-Champion, Adriaan G. Holleboom

**Affiliations:** 1 Radiology and Nuclear Medicine, Amsterdam UMC location University of Amsterdam, Amsterdam, The Netherlands; 2 Vascular Medicine, Amsterdam UMC location University of Amsterdam, Amsterdam, The Netherlands; 3 Amsterdam Gastroenterology Endocrinology Metabolism (AGEM) Institute, Amsterdam UMC, University of Amsterdam, Amsterdam, The Netherlands; 4 Gastroenterology and Hepatology, Amsterdam UMC location University of Amsterdam, Amsterdam, The Netherlands; 5 Perspectum Ltd., Oxford, United Kingdom; 6 Department of Pathology, Erasmus University Medical Center, Rotterdam, The Netherlands; 7 Pathology, Amsterdam UMC location University of Amsterdam, Amsterdam, The Netherlands

**Keywords:** MASLD, MASH, Multiparametric magnetic resonance imaging, Biomarkers, Fibrosis

## Abstract

**Objectives:**

Metabolic dysfunction-associated steatotic liver disease (MASLD) is increasing both in numbers and severity worldwide. Non-invasive alternatives to liver biopsy, particularly for the detection of metabolic dysfunction-associated steatohepatitis (MASH), have proven difficult to establish. We aimed to assess whether quantitative MRI (qMRI) alone and in combination with laboratory and anthropometric measurements and other non-invasive tests (NITs) can detect stages of MASLD.

**Materials and methods:**

In this single-center prospective cohort study, 91 participants with hepatic steatosis on ultrasound or vibration-controlled transient elastography were enrolled in the outpatient clinics between September 2018 and January 2024. Patients underwent blood sampling, qMRI and liver biopsy. Non-invasive parameters were correlated with histopathology in all 91 participants, of whom 37 were reported previously. Prediction models for advanced steatosis (S3), MASH, fibro-MASH (S ≥ 1, lobular inflammation ≥ 1, hepatocyte ballooning ≥ 1 and F ≥ 2), significant (≥ F2) and advanced (≥ F3) fibrosis were designed based on 88 MASLD patients.

**Results:**

MR elastography (MRE)-derived elasticity (MRE-G’), MRE-derived stiffness (MRE-Gabs) and LiverMultiScan® iron-corrected T1 (cT1) correlated with hepatocyte ballooning (Spearman’s R: 0.45 (*p* < 0.001); 0.42 (*p* < 0.001); 0.38 (*p* < 0.001)). Prediction models for ≥ F3 outperformed MAF5 and FIB4, but did not outperform ELF or NFS. A model combining cT1, MRE-G’, aspartate aminotransferase and alanine aminotransferase yielded an AUC of 0.83 (95% CI: 0.74–0.93) for fibro-MASH, not outperforming FibroScan-AST-score (FAST) or cT1-AST-fasting glucose score (cTAG) (*p* = 0.130; *p* = 0.284).

**Conclusion:**

qMRI parameters are able to differentiate degrees of MASLD severity. Generally, the addition of other available measurements did not significantly improve accuracy compared to individual qMRI parameters or established NITs.

**Key Points:**

***Question*** *Refinement of non-invasive tools is needed to accurately stage metabolic-associated steatotic liver disease (MASLD), particularly progressive disease and significant and advanced fibrosis.*

***Findings*** *Quantitative MRI has good diagnostic accuracy to stage MASLD. The combination of MRI parameters with laboratory and anthropometric measurements has limited additional benefit.*

***Clinical relevance*** *This research provides valuable insights for clinicians seeking to reduce reliance on liver biopsy. The findings could be applied in clinical settings to guide earlier, less invasive diagnosis and disease monitoring, allowing for timely interventions and more personalized treatment strategies.*

**Graphical Abstract:**

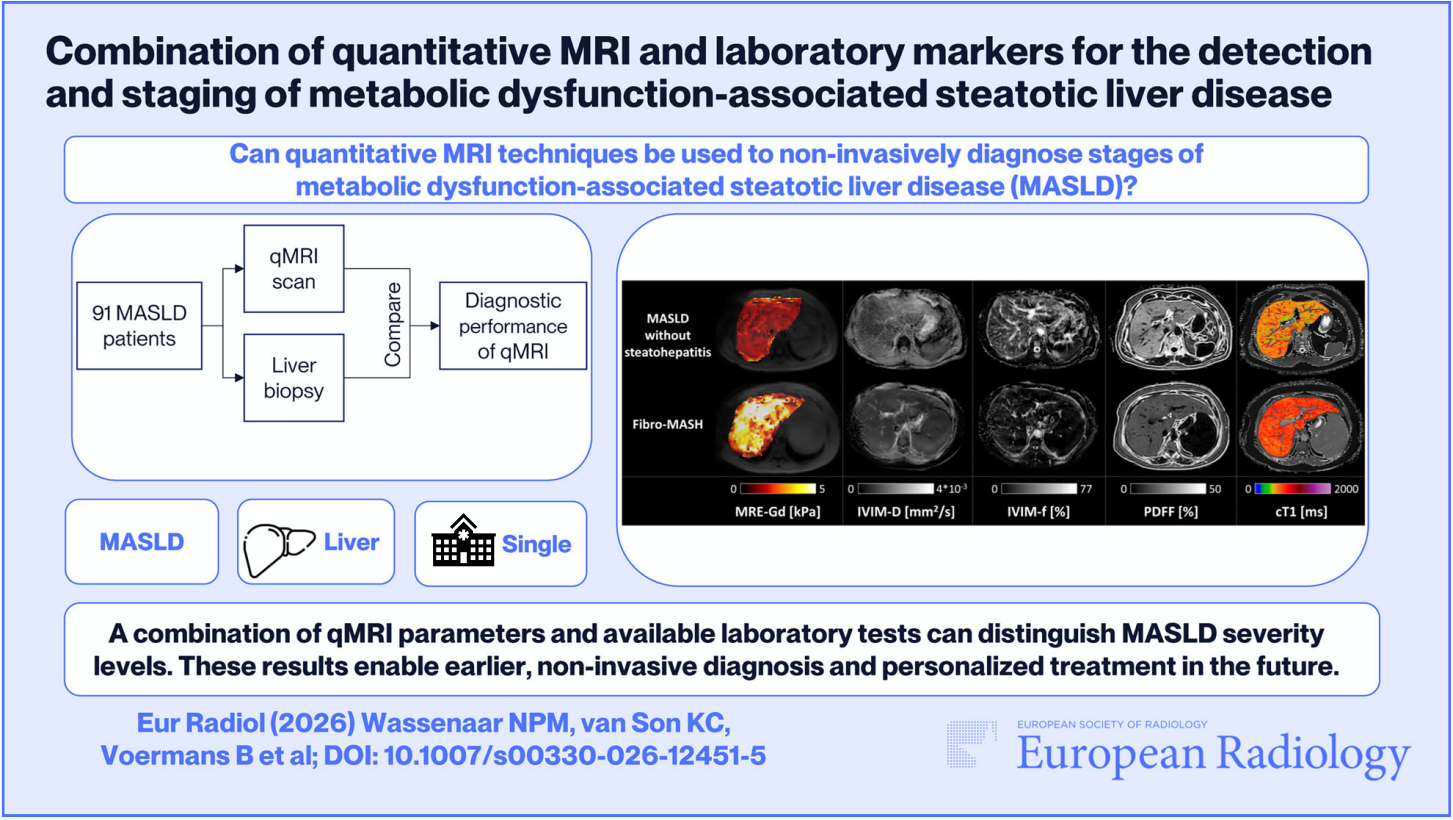

## Introduction

Approximately 30% of adults worldwide have metabolic dysfunction-associated steatotic liver disease (MASLD) and are therefore at increased risk of liver complications (e.g., cirrhosis, hepatocellular carcinoma) and mortality [1]. MASLD is defined as the presence of fat in ≥ 5% of hepatocytes, in the presence of metabolic risk factors, especially type 2 diabetes mellitus (T2DM) and obesity [2], and is the most rapidly increasing contributor to disease burden related to complications of chronic liver disease [1]. The spectrum of disease ranges from isolated steatosis, through metabolic dysfunction-associated steatohepatitis (MASH), to fibrosis and ultimately cirrhosis and hepatocellular carcinoma [2]. The reference standard for diagnosing and staging MASLD is liver biopsy. Yet, liver biopsy has clear drawbacks: it is an invasive [3] and labor-intensive procedure, with possible sampling error [4] and evident interobserver variability [5]. These drawbacks have spurred the development of non-invasive alternatives to diagnose, stage and monitor MASLD.

Quantitative MRI techniques (qMRI) are increasingly recognized as alternatives for liver biopsy as they can non-invasively quantify the underlying tissue characteristics and predict clinical outcomes [6, 7]. MASLD is characterized by an altered metabolism in the liver involving excessive accumulation of free fatty acids [8]. Hepatic steatosis can be quantified with MRI-proton density fat fraction (PDFF) [9]. Inflammation and hepatocyte ballooning distinguish MASH from MASLD without steatohepatitis, making them indicators of disease activity. Previous research showed that increased free water content in inflamed tissue can lead to higher viscosity measured with MR elastography (MRE) and prolonged T1 relaxation times [6, 10]. Diffusivity in the liver may be reduced due to the infiltration of immune cells and can be measured with diffusion-weighted MRI (DWI) [11, 12]. The pro-inflammatory environment in the liver leads to the deposition of extracellular matrix and fibrogenesis [8]. Fibrotic tissue in the liver can be measured with MRE [13]. Moreover, previous studies have found that T1 mapping may capture fibrosis in MASLD [14].

Previous research showed that combining qMRI techniques increases the diagnostic accuracy for diagnosing and staging MASLD [15]. Accuracy may be further enhanced by combining qMRI techniques with other non-invasive biomarkers. As such, the MR-MASH score uses PDFF, waist circumference and height to identify MASH [16], the MRI-aspartate aminotransferase (MAST) score uses PDFF, MRE and aspartate aminotransferase (AST) to identify patients with fibrotic MASH (fibro-MASH) (MASLD activity score (MAS) ≥ 2 and significant fibrosis (≥ F2)) [17], and the MEFIB score uses MRE and the Fibrosis-4 score (FIB4) to identify patients with ≥ F2 [18]. However, a comprehensive comparison of qMRI techniques, laboratory measurements, blood-based biomarker scores, vibration-controlled transient elastography (VCTE) and clinical characteristics with biopsy scores has not yet been conducted. Therefore, this study aimed to assess whether qMRI alone and in combination with laboratory and anthropometric measurements and other noninvasive tests (NITs) can detect stages of MASLD, including fibro-MASH, ≥ F2 and advanced (≥ F3) fibrosis. These outcomes are particularly important as they are associated with higher risks of disease progression, morbidity and mortality [19].

## Materials and methods

### Participants

Data, acquired between September 2018 and January 2024, from the Amsterdam NAFLD-NASH cohort (ANCHOR) study were used. The study is registered in the Dutch Trial Register (NL-OMON52739) and conducted at the Amsterdam UMC, in compliance with the Declaration of Helsinki principles and according to Good Clinical Practice guidelines. Inclusion criteria are hepatic steatosis on abdominal ultrasound or VCTE and age ≥ 18 years [12]. All participants fasted for at least 4 h prior to the study procedures. This study is a second, larger analysis of prospectively collected data from the cohort described by Troelstra et al, of which the previous publication represented an interim analysis including the first 37 patients [12]. All patients underwent laboratory measurements, VCTE examinations and a qMRI scan including PDFF, MRE, intravoxel incoherent motion (IVIM) DWI MRI and iron-corrected T1 (cT1) mapping.

### Laboratory measurements and VCTE

We refer to the Supplementary Material Section 1 for information on the performed laboratory measurements, biomarker analysis and VCTE examinations. Of note, VCTE captures controlled attenuation parameter (VCTE-CAP), a measure of steatosis, and liver stiffness measurement (VCTE-LSM), a measure of fibrosis [20].

### Liver MRI acquisition

All participants underwent a single-session 3-T MRI scan (Ingenia, Philips) using a 16-channel dStream torso and posterior coil. A magnitude-based PDFF scan was conducted with a multi-slice multi-echo gradient echo sequence. MRE scans deployed a fractionally encoded multi-slice gradient echo acquisition [21, 22]. A gravitational transducer, placed on the midaxillary line at the height of the xiphoid process, had a vibrational frequency of 50 Hz. IVIM-DWI MR images were acquired with a free-breathing multi-slice diffusion-weighted single-shot echo-planar sequence with 18 b-values. The LiverMultiScan® protocol (Perspectum Ltd.) was used for cT1 mapping [23]. MRI acquisition parameters can be found in Table S2.

### MRI post-processing

Prominent vessels, bile ducts, liver edges, and image artifacts were avoided in all post-processing steps. A researcher (N.W.) with 4 years of experience in abdominal qMRI placed regions of interest (ROIs) in ITK Snap (v3.8.0; http://www.itksnap.org/) [24]. For PDFF, three circular ROIs were placed in the liver on three different slices of the first echo time image. A multi-echo, multi-frequency water-fat signal model with T2* correction was fitted (Matlab v2021b; The Mathworks Inc.) to the mean signal intensity per echo time [25] to obtain liver fat fraction (PDFF) and T2* value.

Post-processing of MRE data was performed according to Sinkus et al [26]. An ROI in the liver is drawn in the three central slices. Voxels with unreliable viscoelastic properties (non-linearity > 50%) were excluded. The mean stiffness (magnitude of the complex shear modulus, MRE-Gabs), elasticity (storage modulus, MRE-G’), and phase angle (MRE-φ) were calculated.

The diffusion coefficient (IVIM-D), perfusion fraction (IVIM-f), and pseudo-diffusion coefficient (IVIM-D*) were calculated from the IVIM-DWI data using open source IVIM-NET (https://github.com/oliverchampion/IVIMNET) in Python (v3.6; PyTorch) [27, 28]. An ROI, encompassing the entire liver, was defined using a U-Net [29] with manual adjustments (https://github.com/dilaratank/BScThesis-AutoLiverSeg).

The LiverMultiScan® software was used to perform cT1 mapping [23] from MOLLI T1 maps. Anonymized multiparametric MRI data were analyzed off-site by trained image analysts blinded to other data. Whole liver segmentation maps were created using a semi-automatic method. Pixel values from these maps were averaged, and median cT1 values across four slices were reported. Inter-reader variability for quantitative MRI scans is assessed in a subgroup of patients. The methods and results of this subanalysis can be found in Supplementary Material Section 2. Figure S4 gives an overview of the post-processing steps.

### Liver biopsy

Percutaneous ultrasound-guided liver biopsies were performed according to the local standard procedure. Histological specimens were read in tandem by two expert pathologists (M.D., J.V.) who were blinded to other data. Histopathological parameters were defined with the use of the steatosis, activity (including lobular inflammation and hepatocyte ballooning) and fibrosis (SAF-) score, classifying non-MASLD, MASLD without steatohepatitis and MASH [30], steatosis grade and fibrosis stage. Fibro-MASH was defined as the presence of steatosis (≥ S1) and disease activity, which was defined as the presence of lobular inflammation (≥ 1), hepatocyte ballooning (≥ 1), and ≥ F2 [30].

### Statistical analysis

Statistical analyses were conducted in RStudio (v4.3.2). *p*-values < 0.05 were considered significant. Spearman’s Rank correlations were computed between SAF-score parameters and qMRI parameters, laboratory measurements, blood-based biomarker scores, VCTE parameters and clinical characteristics. The ability to differentiate between SAF-score parameters was tested using the one-way ANOVA test with post hoc Tukey HSD test or the Kruskal–Wallis test with post hoc Dunn’s test with Bonferroni correction for normally and non-normally distributed data, respectively. In case of missing data, data were imputed using the MICE package in RStudio with predictive mean matching (PMM) using all parameters. Trace plots and boxplots comparing the observed vs. missing data were employed to assess the validity and robustness of imputation.

Univariate logistic regression models were created to detect S3, MASH, fibro-MASH, ≥ F2 and ≥ F3 separately using 5-fold cross-validation on the abovementioned parameters in those with histologically proven MASLD (≥ S1). Model performance was measured using the area under the receiver operating characteristic curve (AUC) (± SD). Subsequently, multivariate logistic regression models were created using 5-fold cross-validation, including the two qMRI parameters with the highest individual AUC (both solely and in combination with each other) and laboratory measurements and clinical characteristics. The model with the highest AUC was selected; if equal, the score easiest to apply in clinical practice was reported. Diagnostic performance of the combined scores was calculated at rule-in and rule-out cut-offs set at 90% sensitivity and 90% specificity. Finally, the diagnostic performance of the composite score was computed and compared to other NITs, i.e., FibroScan-AST (FAST) [31], NAFLD fibrosis score (NFS), metabolic dysfunction-associated fibrosis-5 score (MAF5) [32], MR-MASH [16], cT1-AST-fasting glucose score (cTAG) [33] and FIB4, using DeLong tests.

## Results

### Participant characteristics

**Fig. 1:**
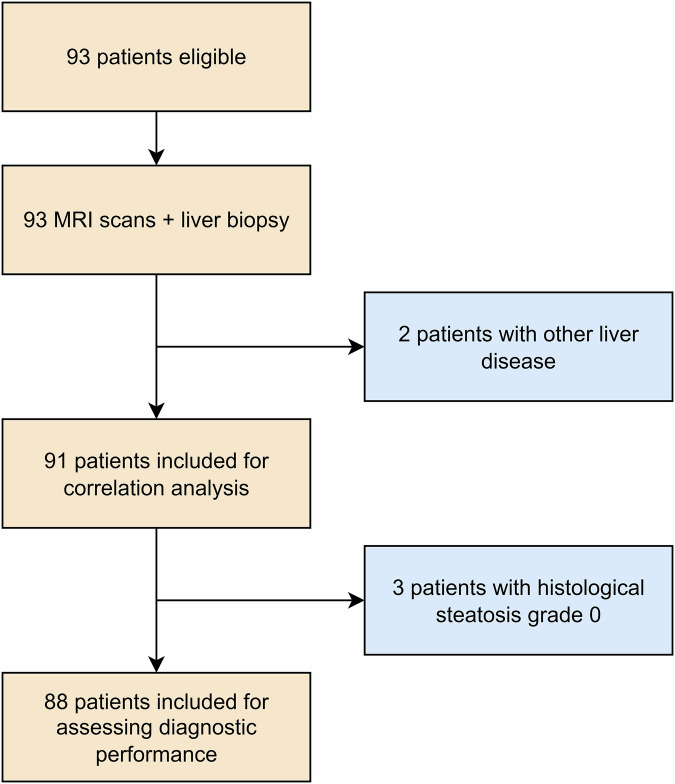
Flow diagram showing the inclusion and exclusion process of participants in the ANCHOR study and participants included in this study

A total of 91 participants were included in the study for correlation analysis with histopathology, of whom 3 had S0. The final MASLD cohort included 88 participants, of whom 27.3% had S3, 69.3% met the criteria for fibro-MASH [30], and 39.8% had ≥ F3 (Table 1, Fig. 1). Table 1 summarizes clinical, imaging, and histology data.

**Table 1:** Clinical characteristics, laboratory measurements, blood-based biomarker scores, VCTE and qMRI parameters and histological assessment of liver biopsy of the study participants

	MASLD cohort (*n* = 88)				
Clinical characteristics					
Age (years)	47.7 (± 14.0)				
Male sex, *n* (%)	54 (61.4)				
BMI (kg/m^2^)	32.98 (29.47–36.67)				
T2DM, *n* (%)	39 (44.3)				
Waist circumference (cm)	114.6 (± 14.0)				
Hip circumference (cm)	114.4 (± 12.3)				
Hypertension, *n* (%)	33 (37.5)				
Laboratory measurements					
AST (U/L)	40.0 (35.0–53.3)				
ALT (U/L)	60.0 (47.5–81.3)				
γGT (U/L)	62.0 (36.0–94.3)				
Platelets (10^9^)	246.6 (± 56.7)				
Fasting glucose (mmol/L)	6.3 (5.4–7.6)				
HbA1c (mmol/mol)	41.5 (36.0–49.3)				
Blood-based biomarker scores					
FIB4	1.06 (0.75-1.59)				
NFS	−1.57 (± 1.52)				
APRI	0.44 (0.34–0.64)				
MAF5	3.72 (2.59–5.12)				
ELF	9.09 (± 0.92)				
VCTE parameters					
VCTE-CAP (dB/m)	341 (311–368)				
VCTE-LSM (kPa)	9.5 (7.6–12.5)				
qMRI parameters					
PDFF (%)	17.51 (± 8.46)				
T2* (ms)	20.21 (± 4.51)				
cT1 (ms)	901.2 (± 105.8)				
MRE-Gabs (kPa)	2.04 (1.87–2.40)				
MRE-G’ (kPa)	1.76 (1.61–2.05)				
MRE-φ (rad)	0.27 (0.24–0.31)				
IVIM-D (10^−^^3^ mm^2^/s)	1.14 (1.08–1.23)				
IVIM-f (%)	15.10 (13.69–18.59)				
IVIM-D* (mm^2^/s)	0.139 (0.128–0.147)				
Histological assessment of liver biopsy using SAF-score	0	1	2	3	4
Steatosis grade, *n* (%)	0	31 (35.2)	33 (37.5)	24 (27.3)	NA
Disease activity, *n* (%)	3 (3.4)	22 (25.0)	44 (50)	17 (19.3)	2 (2.3)
Lobular inflammation, *n* (%)	4 (4.5)	77 (87.5)	7 (8.0)	NA	NA
Hepatocyte ballooning, *n* (%)	25 (28.4)	48 (54.5)	15 (17.0)	NA	NA
Fibrosis score, *n* (%)	3 (3.4)	10 (11.4)	40 (45.5)	27 (30.7)	8 (9.1)
MASH, *n* (%)	62 (70.5)				
Fibro-MASH, *n* (%)	61 (69.3)				

*BMI* body mass index, *T2DM* type 2 diabetes mellitus, *AST* aspartate transferase, *ALT* alanine transferase, *γGT* gamma-glutamyltransferase, *HbA1c* glycated hemoglobulin, *FIB4* fibrosis-4 score, *NFS* non-alcoholic fatty liver fibrosis score, *APRI* AST to platelet ratio index, *MAF5* metabolic dysfunction-associated fibrosis-5 score, *ELF* enhanced liver fibrosis score, *VCTE* vibration-controlled transient elastography, *CAP* controlled attenuation parameter, *LSM* liver stiffness measurement, *PDFF* proton density fat fraction, *cT1* iron-corrected T1, *MRE* MR elastography, *Gabs* stiffness, *G’* elasticity, *φ* phase angle, *IVIM* intravoxel incoherent motion, *D* diffusion coefficient, *f* perfusion fraction, D* pseudo-diffusion coefficient

**Fig. 2:**
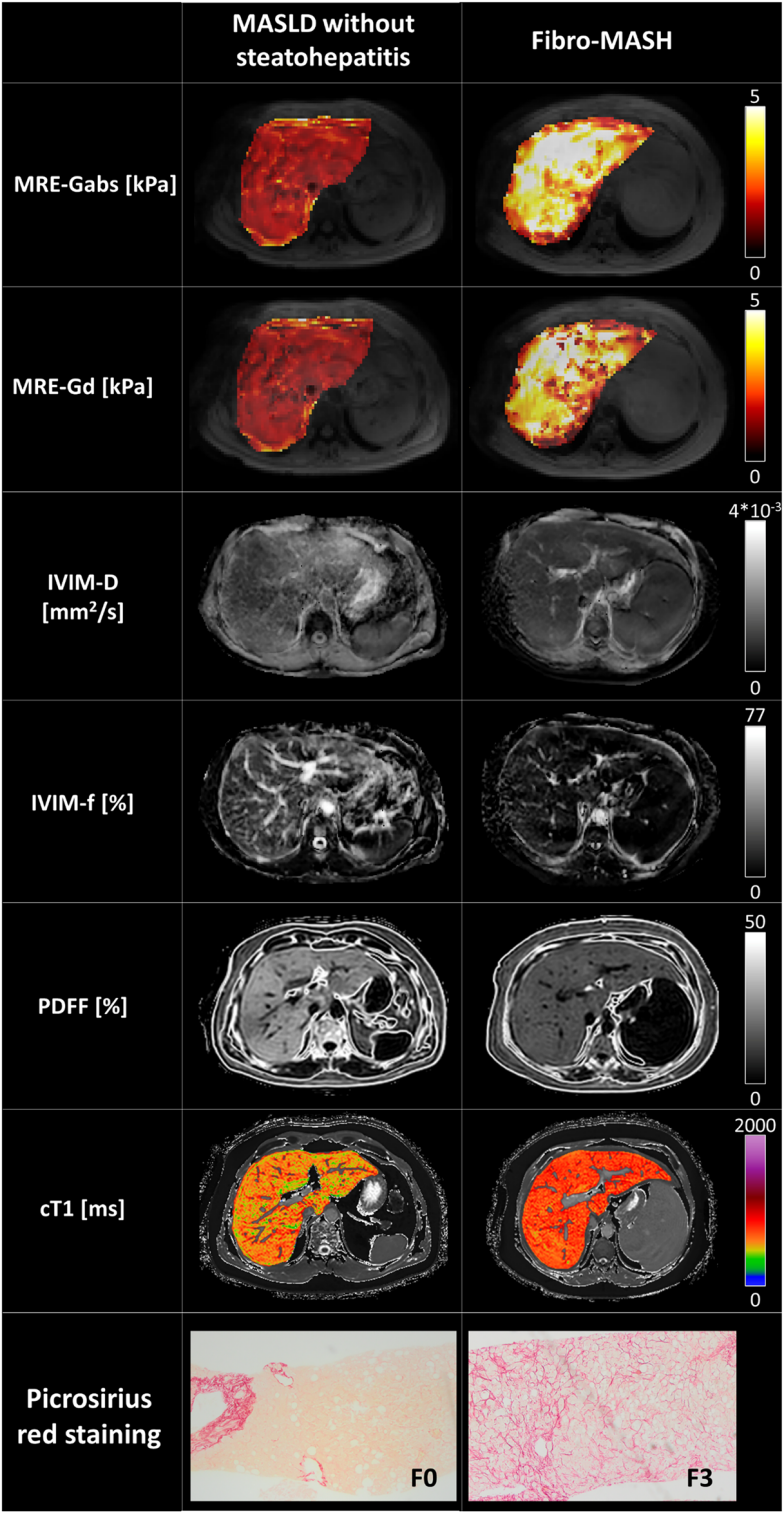
Parameter maps for MRE-Gabs, MRE-G’, IVIM-D, IVIM-f, PDFF and cT1 shown for a 44-year-old man with MASLD without steatohepatitis (S1, lobular inflammation 1, hepatocyte ballooning 0, F0), and a 44-year-old woman with fibro-MASH (S3, lobular inflammation 2, hepatocyte ballooning 2, F3). A histology image stained with picrosirius red, used to assess fibrosis stage, is also included. MASLD, metabolic dysfunction-associated steatotic liver disease; MASH, metabolic dysfunction-associated steatohepatitis; MRE, MR elastography; Gabs, stiffness; G’, elasticity; IVIM, intravoxel incoherent motion; D, diffusion; f, perfusion fraction; PDFF, proton density fat fraction; cT1, iron-corrected T1

Figure 2 shows representative scans of two patients: one with MASLD without steatohepatitis and one with MASH. Due to technical or time-related issues, some participants did not have the full set of qMRI scan parameters recorded: five MRE scans, five IVIM-DWI scans, seven LiverMultiScans® and five PDFF scans were missing. MRE quality was considered insufficient in eight participants, and cT1 was not quantifiable in seven participants. A missing data plot can be seen in Fig. S5.

### Correlations with histopathology

Eight variables correlated with histological steatosis (Fig. S6). Among them, PDFF showed the highest correlation (Spearman’s R = 0.66, *p* < 0.001), followed by cT1 (Spearman’s R = 0.62, *p* < 0.001) (Fig. S7A, B).

Seventeen variables correlated with hepatocyte ballooning (Fig. S6). MRE-G’ showed the highest Spearman’s R of 0.45 (*p* < 0.001), followed by MRE-Gabs with a Spearman’s R of 0.42 (*p* < 0.001) and cT1 with a Spearman’s R of 0.38 (*p* < 0.001). MRE-G’ distinguished between scores for hepatocyte ballooning (0–1: *p* = 0.005; 0–2: *p* < 0.001; 1–2: *p* = 0.007) (Fig. S7C) while cT1 distinguished between no hepatocyte ballooning (score 0) and any hepatocyte ballooning (score ≥ 1) (0–1: *p* < 0.001, 0–2: *p* = 0.001) (Fig. S7D). Although lobular inflammation correlated significantly with two other histological parameters, i.e., hepatocyte ballooning (Spearman’s R: 0.24 (*p* = 0.023)) and fibrosis (Spearman’s R: 0.22 (*p* = 0.039)), no significant associations with other non-invasive markers were observed (Fig. S6).

Sixteen noninvasive markers correlated to histological fibrosis, of which MRE-G’ and FIB4 showed the strongest correlation, both with a Spearman’s R of 0.54 (*p* < 0.001) (Figs. S6, S7E, F).

### Derivation of new composite scores

Univariate logistic regression using 5-fold cross-validation showed that the presence of fibro-MASH was best detected by MAF5 (AUC: 0.76 (± 0.13)), followed by MRE-G’ (0.73 (± 0.14)) and cT1 (AUC: 0.72 (± 0.04)) (Tables 2, S3). A model combining cT1, MRE-G’, AST and ALT had the highest AUC (AUC: 0.81 (± 0.12)): $\log \frac{{{{\rm{P}}}}\left({\mbox{Fibro}}-{\mbox{MASH}}\right)}{1-{{{\rm{P}}}}\left({\mbox{Fibro}}-{\mbox{MASH}}\right)}=-8.914+0.006* {{{\rm{cT}}}}1+1.458* {\mbox{MRE}}-{\mbox{G}}{\prime} +0.040* {{{\rm{AST}}}}-0.007* {{{\rm{ALT}}}}$. At the rule-in threshold set at 90% sensitivity, the combined score had a specificity of 0.37 and a positive and negative predictive value of 0.76 and 0.63, respectively (Table S5).

**Table 2:** AUC (± SD) of the top five parameters with the highest AUC to differentiate between different histologically proven stages of MASLD (fibro-MASH; < F2 vs. ≥ F2; < F3 vs. ≥ F3) and the composite score using 5-fold cross-validation

Stage of MASLD	Parameter	AUC (± SD)
Fibro-MASH	MAF5	0.76 (0.13)
	MRE-G’	0.73 (0.14)
	cT1	0.72 (0.04)
	AST	0.71 (0.13)
	MRE-Gabs	0.70 (0.21)
	Composite score	0.81 (0.12)
< F2 vs. ≥ F2	MAF5	0.87 (0.11)
	VCTE-LSM	0.83 (0.09)
	AST	0.81 (0.21)
	NFS	0.76 (0.21)
	APRI	0.76 (0.09)
	Composite score	0.90 (0.14)
< F3 vs. ≥ F3	NFS	0.80 (0.14)
	MRE-G’	0.80 (0.10)
	VCTE-LSM	0.78 (0.19)
	ELF	0.76 (0.05)
	MRE-Gabs	0.75 (0.13)
	Composite score	0.82 (0.07)

*AUC* area under the curve, *SD* standard deviation, *MASH* metabolic dysfunction-associated steatohepatitis, *F2* significant fibrosis, *F3* advanced fibrosis, *AST* aspartate transferase, *ALT* alanine aminotransferase, *FIB4* fibrosis-4 score, *NFS* non-alcoholic fatty liver fibrosis score, *MAF5* metabolic dysfunction-associated fibrosis-5 score, *LSM* vibration-controlled transient elastography derived liver stiffness measurement, *PDFF* proton density fat fraction, *cT1* iron-corrected T1, *G’* MR elastography derived elasticity

Univariate logistic regression revealed that < F2 vs. ≥ F2 was best differentiated by MAF5 (AUC: 0.87 (± 0.11)), followed by VCTE-LSM (AUC: 0.83 (± 0.09)) and AST (AUC: 0.81 (± 0.21)) (Tables 2, S3). A model combining MRE-G’, ALT and waist circumference had the highest AUC (0.90 (± 0.14)): $\log \frac{{{{\rm{P}}}}\left( < {{{\rm{F}}}}2\right)}{1-{{{\rm{P}}}}\left( < {{{\rm{F}}}}2\right)}=-12.539+2.957* {\mbox{MRE}}-{\mbox{G}}{\prime} +0.010* {{{\rm{ALT}}}}+0.076* {{{\rm{waist\; circumference}}}}$.

Univariate logistic regression revealed that < F3 vs. ≥ F3 was best differentiated by NFS (AUC: 0.80 (± 0.14)), followed by MRE-G’ (AUC: 0.80 (± 0.10)) and VCTE-LSM (AUC: 0.78 (± 0.19)) (Tables 2, S3). A model combining MRE-G’, AST, ALT and waist circumference had the highest AUC (0.82 (± 0.07)): $\log \frac{{{{\rm{P}}}}\left( < {{{\rm{F}}}}3\right)}{1-{{{\rm{P}}}}\left( < {{{\rm{F}}}}3\right)}=-8.761+2.464* {\mbox{MRE}}-{\mbox{G}}{\prime} +0.051* {{{\rm{AST}}}}-0.043* {{{\rm{ALT}}}}+0.035* {{{\rm{waist\; circumference}}}}$.

The results for detecting < 3 vs. S3 and MASH and results for univariate and multivariate logistic regression using the complete dataset (*n* = 51)are shown in the Supplementary Material Section 3.

### Diagnostic performance of new composite scores versus other NITs

**Fig. 3:**
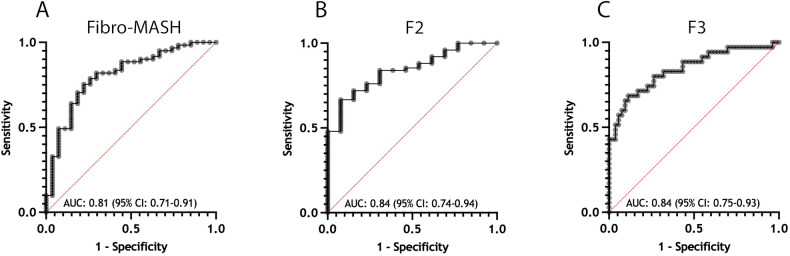
ROC curves for the composite scores and other NITs for the differentiation of fibro-MASH (**A**), < F2 vs. ≥ F2 (**B**), and < F3 vs. ≥ F3 (**C**). The AUC with 95% CI is stated in the figure

When computed in the MASLD cohort (*n* = 88 participants), the composite score for fibro-MASH had an AUC (0.81 (95% CI: 0.71–0.91)) (Fig. 3, Table 3). AUC of MAF5, cTAG, and FAST for diagnosing fibro-MASH were 0.76 (95% CI: 0.66–0.87 (*p* = 0.441)), 0.75 (95% CI: 0.63–0.88 (*p* = 0.284)) and 0.72 (95% CI: 0.59–0.84 (*p* = 0.130)), respectively.

**Table 3:** AUC (95% CI) of the composite scores and other NITs for the differentiation between different histologically proven stages of MASLD (fibro-MASH; < F2 vs. ≥ F2; < F3 vs. ≥ F3) and comparison of AUC of the composite score with established NITs using DeLong test

Stage of MASLD	Parameter	AUC (95% CI)	*p*-value (compared to composite score)
Fibro-MASH	Composite score	0.83 (0.74–0.93)	-
	cT1	0.72 (0.60–0.85)	0.073
	FAST	0.72 (0.59–0.84)	0.130
	MAF5	0.76 (0.66–0.87)	0.441
	MRE-G’	0.73 (0.62–0.84)	0.172
	cTAG	0.75 (0.63–0.88)	0.284
< F2 vs. ≥ F2	Composite score	0.84 (0.74–0.94)	-
	MAF5	0.87 (0.78–0.96)	0.530
	VCTE-LSM	0.82 (0.71–0.94)	0.792
	NFS	0.78 (0.65–0.91)	0.453
	AST	0.79 (0.64–0.94)	0.559
	MRE-G’	0.75 (0.62–0.89)	0.149
< F3 vs. ≥ F3	Composite score	0.84 (0.75–0.93)	-
	MRE-G’	0.78 (0.69–0.88)	0.168
	NFS	0.79 (0.69–0.89)	0.210
	VCTE-LSM	0.78 (0.68–0.88)	0.198
	ELF	0.75 (0.64–0.87)	0.122
	MAF5	0.73 (0.62–0.85)	**0.031**
	FIB4	0.73 (0.62–0.84)	**0.025**

Significant *p*-values are highlighted in bold

*AUC* area under the curve, *CI* confidence interval, *MASH* metabolic dysfunction-associated steatohepatitis, *F2* significant fibrosis, *F3* advanced fibrosis, *cT1* iron-corrected T1, *VCTE-LSM* vibration-controlled transient elastography derived liver stiffness measurement, *FAST* FibroScan-AST score, *NFS* non-alcoholic fatty liver fibrosis score, *MRE* magnetic resonance elastography, *MRE-G’* MR elastography derived elasticity, *ELF* enhanced fibrosis test, *MAF5* metabolic dysfunction-associated fibrosis-5 score, *FIB4* fibrosis-4 score, *cTAG* cT1-AST-fasting glucose score

The composite score for ≥ F2 had an AUC of 0.84 (95% CI: 0.74–0.94) (Fig. 3) in the study cohort compared to 0.87 (95% CI: 0.78–0.96) (*p* = 0.530) and 0.82 (95% CI: 0.71–0.94) (*p* = 0.792) for MAF5 and VCTE-LSM, respectively (Table 3).

The composite score for ≥ F3 had an AUC of 0.84 (95% CI: 0.75–0.93) (Fig. 3), outperforming MAF5 (0.73 (95% CI: 0.62–0.85) (*p* = 0.031)) and FIB4 (0.73 (95% CI: 0.62–0.84) (*p* = 0.025)). The composite score for ≥ F3 did not outperform MRE-G’ 0.78 (95% CI: 0.69–0.88 (*p* = 0.168)), NFS 0.79 (95% CI: 0.69–0.89 (*p* = 0.210)), VCTE-LSM (0.78 (95% CI: 0.68–0.88) (*p* = 0.198)) and ELF (0.75 (95% CI: 0.64–0.87) (*p* = 0.123)) (Table 3). The AUC of the composite score for S3 and MASH compared to other NITs can be seen in Table S6. Figures S8–S12 show the ROC curves for the composite scores and NITs for all outcomes.

## Discussion

This study showed that an extensive panel of qMRI parameters can accurately diagnose different stages of MASLD severity. Prediction models for ≥ F3, combining MRE-G’, AST, ALT and waist circumference, significantly outperformed MAF5 and FIB4, but, although higher, did not significantly outperform ELF or NFS. Prediction models for fibro-MASH and ≥ F2 failed to demonstrate a significant increase in diagnostic accuracy compared to other NITs. Previous research focused on the role of qMRI in diagnosing different stages of MASLD severity. However, a comprehensive comparison of qMRI techniques, laboratory measurements, blood-based biomarker scores, vibration-controlled transient elastography (VCTE) and clinical characteristics with biopsy scores has not yet been conducted.

**Fig. 4:**
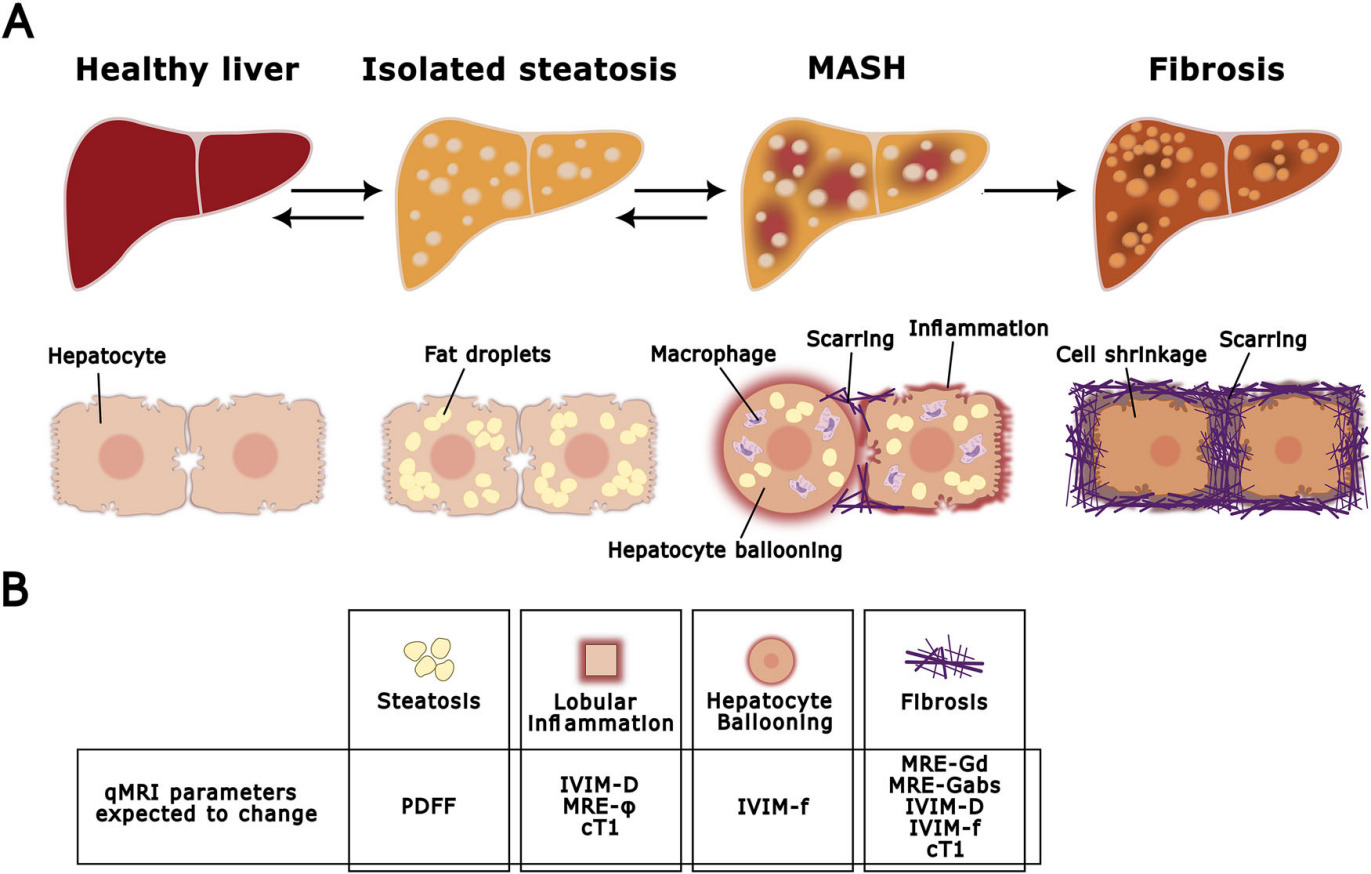
Histopathological changes along the disease spectrum of MASLD (**A**) and quantitative MRI parameters that are expected to change for the individual histopathological components (**B**). MASH, metabolic dysfunction-associated steatohepatitis; qMRI, quantitative MRI; PDFF, proton density fat fraction; IVIM, intravoxel incoherent motion; D, diffusion; MRE, MR elastography; φ, phase angle; cT1, iron-corrected T1; f, perfusion fraction; Gd, elasticity; Gabs, stiffness

Our study builds upon previous efforts to develop non-invasive models to detect fibro-MASH, most notably the FAST- and MAST-scores, and cTAG [17, 31, 33]. FAST is comprised of VCTE-LSM, VCTE-CAP and AST and demonstrated satisfactory to excellent performance in validation cohorts (AUC: 0.74–0.94). MAST is comprised of PDFF, MRE and AST and demonstrated good performance for the detection of fibro-MASH in validation cohorts (AUC: 0.93). Unfortunately, validation of MAST was not possible in our cohort due to the use of different MRE techniques, preventing head-to-head comparison. MAST and FAST both include a measure for steatosis and fibrosis, accompanied by AST. cTAG is comprised of cT1, AST and fasting glucose and showed an AUC of 0.90 for the detection of fibro-MASH in previous research [33]. Our composite score for the detection of fibro-MASH is comprised of parameters that directly reflect underlying pathophysiological processes in the liver (Fig. 4); increased free water content in inflamed tissue leads to prolonged T1 relaxation times, and MRE-G’ is increased due to the fibrotic tissue in the liver [6, 10, 34]. In head-to-head comparisons with FAST and cTAG, our composite score for fibro-MASH did not demonstrate significantly improved accuracy in this cohort.

For staging fibrosis, stiffness assessed by MRE or VCTE has increasingly gained acceptance in clinical practice. MRE-G’ and VCTE-LSM showed good diagnostic performance for the detection of ≥ F2 and ≥ F3. The composite scores identified in this study consisted of MRE-G’, ALT, and waist circumference for ≥ F2, with the addition of AST for ≥ F3. These composite scores did not outperform the stiffness measures or other more readily available non-MRI NITs, with the exception of MAF5 and FIB4 in detecting ≥ F3.

The tested parameters correlated with multiple histopathological features included in the SAF-score. This may be explained by the interdependence of these scores and the pathophysiological development of MASLD. Lipid droplet accumulation and lipotoxic injury cause swelling of the hepatocytes, and this prolonged liver injury leads to the activation of hepatic stellate cells, the main cell type responsible for progressive fibrogenesis [8]. Hepatocyte ballooning primarily differentiates MASH and isolated steatosis in this cohort, as lobular inflammation shows limited variability. Interestingly, the composite scores do not merely include the top four individual parameters with the highest AUC. This displays the redundancy of some combination of variables; i.e., MRI-G’ and MRI-Gabs, representing the same pathophysiological process, are not selected within the same model.

The ANCHOR cohort represents a population encompassing the full spectrum of MASLD. It captures a metabolically challenged group with a high prevalence of obesity and T2DM, thereby accurately representing the target population for screening and treatment interventions for fibrotic MASLD. A large set of qMRI parameters and other NITs, including both more novel and clinically established ones, were used in this study, thus improving clinical and future usability. Given the extensive range of parameters considered for the detection of different stages of MASLD, this approach allows us to comprehensively investigate diagnostic markers and their relevance across the disease spectrum. The use of a 5-fold cross-validation model provided partial internal validation of the models and a realistic estimate of AUC, sensitivity and specificity. The inter-reader variability analysis (Supplementary Material Section 2) demonstrates good to excellent agreement across all qMRI techniques evaluated. This level of consistency between readers is essential for the reliable clinical adoption of these imaging methods.

This study has some limitations. First, the use of the same cohort to derive the composite scores and to test their performance may have led to an overestimation of the diagnostic performance of the newly derived composite scores compared to other NITs. External validation in an independent cohort is desirable to confirm the generalizability of our findings. Second, the reproducibility of the used qMRI techniques is not assessed in this study, and only one observer delineated the regions of interest. Previous studies have reported on the reproducibility of qMRI scans, which should be considered when implementing these techniques [35–37]. Third, some qMRI scans were missing due to technical or image-quality issues, which may lead to bias in diagnostic accuracy estimates. Data was imputed using PMM, which relies on several assumptions. It assumes that data are missing at random, meaning that missingness can be explained by observed variables included in the imputation model, which was the case in this dataset. PMM also relies on the availability of similar observed cases, and therefore performs best when the missing values fall within the distribution of the observed data. There remains a risk that the results presented in this study are influenced by the assumptions of the imputation model and the limited cohort size. Consequently, the impact of imputing missing data on diagnostic performance cannot be fully excluded. To ensure the robustness of our findings, validation in larger, independent cohorts is required. Fourth, liver biopsy has several drawbacks, including interobserver variability, sampling error [3, 4] and reliance on categorical histological scores that obscure the continuous nature of disease activity. Despite these drawbacks, biopsy assessed with the SAF scoring system remains the reference standard for diagnosing and staging MASLD. Therefore, qMRI techniques must first demonstrate non-inferiority to liver biopsy before clinical implementation. Fifth, logistic regression analysis is currently performed to differentiate between dichotomized clinically relevant outcomes. However, this does not capture the full spectrum of fibrosis severity, and ordinal regression could have allowed a more comprehensive assessment across all stages. Lastly, although the composite scores occasionally demonstrated higher AUCs than other NITs, these differences did not reach statistical significance, which may in part be due to the limited sample size of the study.

This study demonstrates that multiple qMRI techniques, including PDFF, cT1, IVIM-f and MRE-G’, correlate with histopathological features across the disease spectrum of MASLD. Furthermore, composite scores combining qMRI parameters with readily available laboratory and anthropometric measurements accurately detect various stages of MASLD, including the key target conditions fibro-MASH and ≥ F2 and ≥ F3. They showed no consistent statistical advantage over individual qMRI parameters or NITs, yet their higher AUCs suggest added discriminative value. External validation in large, independent cohorts is warranted to evaluate the specific role and optimal application of qMRI parameters, thereby facilitating their future integration into clinical care pathways for non- or minimally invasive detection of patients with progressive disease.

## Supplementary information

ELECTRONIC SUPPLEMENTARY MATERIAL

## Abbreviations

ALT: Alanine aminotransferase
ANCHOR: Amsterdam NAFLD-NASH
AST: Aspartate aminotransferase
AUC: Area under the receiver operating characteristic curve
CI: Confidence interval
cT1: Iron-corrected-T1
F2: Significant fibrosis
F3: Advanced fibrosis
FAST: FibroScan-AST
FIB4: Fibrosis-4
Fibro-MASH: Fibrotic MASH
G’: Elasticity
Gabs: Stiffness
IVIM: Intravoxel incoherent motion
IVIM-D: Intravoxel incoherent motion-derived diffusion coefficient
IVIM-D*: Intravoxel incoherent motion-derived pseudo-diffusion coefficient
IVIM-f: Intravoxel incoherent motion-derived perfection fraction
LSM: Liver stiffness measurement
MAF5: Metabolic dysfunction-associated fibrosis-5 score
MASH: Metabolic dysfunction-associated steatohepatits
MASLD: Metabolic dysfunction-associated steatotic liver disease
MAST: MRI-aspartate aminotransferase
MRE: MR elastography
NFS: NAFLD fibrosis score
NITs: Non-invasive tests
PDFF: Proton density fat fraction
qMRI: Quantitative MRI
ROI: Region of interest
S3: Advanced steatosis
SAF: Steatosis activity fibrosis
VCTE: Vibration-controlled transient elastography
φ: Phase angle
