# Supplementary material for: Combination of quantitative MRI and laboratory markers for the detection and staging of metabolic dysfunction-associated steatotic liver disease
Source: Eur Radiol. 2026 Mar 28;36(8):6523–34. doi: 10.1007/s00330-026-12451-5 (PMC13342414; doi:10.1007/s00330-026-12451-5)
Supplement: Supplementary file 1 — ELECTRONIC SUPPLEMENTARY MATERIAL [file 330_2026_12451_MOESM1_ESM.pdf]

Combination of Quantitative MRI and Laboratory Markers for the Detection and Staging of Metabolic Dysfunction-Associated Steatotic Liver Disease
ELECTRONIC SUPPLEMENTARY MATERIAL

1. Supplemental methods

Laboratory measurements and biomarker analysis

Laboratory measurements (AST, ALT, gamma-glutamyl transpeptidase (γGT), platelets, fasting glucose and glycated hemoglobulin) were collected and analyzed according to local protocol. Blood-based biomarker scores (FIB4, NAFLD fibrosis score (NFS), AST to Platelet Ratio Index (APRI), Metabolic Dysfunction–Associated Fibrosis 5 score (MAF5)) (1) were calculated. Serum, heparinized plasma and EDTA plasma samples were stored in a designated biobank. ELF was determined in serum biobank samples at our endocrine laboratory using kits provided by Siemens^{TM} according to manufactures' instructions using the Atellica IM analyser (Siemens Heathineers).

VCTE examinations

VCTE examinations were performed by experienced physicians (BLINDED) prior to the MRI scan and liver biopsy. A FibroScan® 530 Compact (Echosens, France) device equipped with both the M- and XL-probe was used. The type of probe used depended on the real-time assessment of the skin-to-liver capsule distance for each participant. VCTE reports controlled attenuation parameter (VCTE-CAP) as a measure of steatosis and liver stiffness measurement (VCTE-LSM) as a measure of fibrosis (19).

References

1. van Kleef LA, Francque SM, Prieto-Ortiz JE, Sonneveld MJ, Sanchez-Luque CB, Prieto-Ortiz RG, et al. Metabolic Dysfunction-Associated Fibrosis 5 (MAF-5) Score Predicts Liver Fibrosis Risk and Outcome in the General Population With Metabolic Dysfunction. Gastroenterology. 2024;167(2):357-67.e9.
2. Eddowes PJ, Sasso M, Allison M, Tsochatzis E, Anstee QM, Sheridan D, et al. Accuracy of FibroScan Controlled Attenuation Parameter and Liver Stiffness Measurement in Assessing Steatosis and Fibrosis in Patients With Nonalcoholic Fatty Liver Disease. Gastroenterology. 2019;156(6):1717-30.

2. Inter-reader variability

Methods

The inter-reader variability of PDFF, T2*, IVIM-D, IVIM-f, IVIM-D*, MRE-G', MRE-Gabs and MRE-φ was determined in a subgroup of 20 patients. Each quantitative MRI scan was delineated by two readers. As cT1 was performed by a third party, for which ICC has been well documented, the ICC for cT1 was taken from literature (Mojtahed et al.)[1].

Inter-reader agreement for quantitative MRI parameters was assessed using the intraclass correlation coefficient (ICC) and Bland-Altman analysis. The ICC was calculated, reflecting the reliability of measurements between the two readers. ICC values were interpreted as follows: values below 0.50 indicate poor agreement, 0.50–0.75 moderate, 0.75–0.90 good, and above 0.90 excellent agreement. The 95% confidence intervals for the ICC were also reported.

In addition, Bland-Altman plots were constructed to visualize the agreement between the two readers. For each parameter, the mean and difference of the paired measurements were calculated and plotted. The mean difference (bias) and the limits of agreement (mean difference ± 1.96 × standard deviation of the differences) were determined to assess systematic bias and the range of inter-reader variability. All statistical analyses were performed using R (version 4.4.1).

Results

The ICC of all parameters are shown in Supplementary table S1. MRE-φ shows good agreement between the two readers. All other parameters show excellent agreement between the two readers. The Bland-Altman plots are shown in Supplementary figures S1-S3.

**Parameter**	**ICC**	**95% CI**	**p-value**	**Bias**	**LoA [lower, upper]**
MRE-Gabs	0.966	0.917 – 0.986	4.95e-13	-0.024	[-0.426, 0.378]
MRE-G'	0.977	0.944 – 0.991	5.03e-15	-0.028	[-0.322, 0.267]
MRE-φ	0.838	0.635 – 0.933	1.5e-06	-0.001	[-0.069, 0.068]
PDFF	0.995	0.989 – 0.998	4.68e-21	0.027	[-1.366, 1.421]
*T2**	0.964	0.912 – 0.986	1.5e-12	0.046	[-2.423, 2.514]
cT1 [1]	*0.93*	*0.89 – 0.97*	*NA*	*0*	*[-32, 31]*
IVIM-D	0.987	0.968 – 0.995	1.36e-17	-0.006	[-0.048, 0.036]
IVIM-f	0.981	0.954 – 0.993	1.68e-15	-0.095	[-1.704, 1.514]
*IVIM-D**	0.918	0.806 – 0.966	1.97e-09	-0.001	[-0.011, 0.010]

Supplementary table S1: Intraclass correlation coefficient (ICC) with 95% confidence intervals (CI) and p-values. All parameters show excellent agreement except for MRE-φ which gives a good agreement between the two readers. The bias with lower and upper limits of agreement (LoA) are also shown.

Abbreviations: PDFF = proton density fat fraction; MRE = MR elastography; Gabs = stiffness; G' = elasticity; φ = phase angle; IVIM = intravoxel incoherent motion diffusion-weighted imaging; D = diffusion coefficient; f = perfusion fraction; D* = pseudo-diffusion coefficient.

References:

1. Mojtahed, A., Kelly, C.J., Herlihy, A.H. et al. Reference range of liver corrected T1 values in a population at low risk for fatty liver disease—a UK Biobank sub-study, with an appendix of interesting cases. Abdom Radiol 44, 72–84 (2019). https://doi.org/10.1007/s00261-018-1701-2

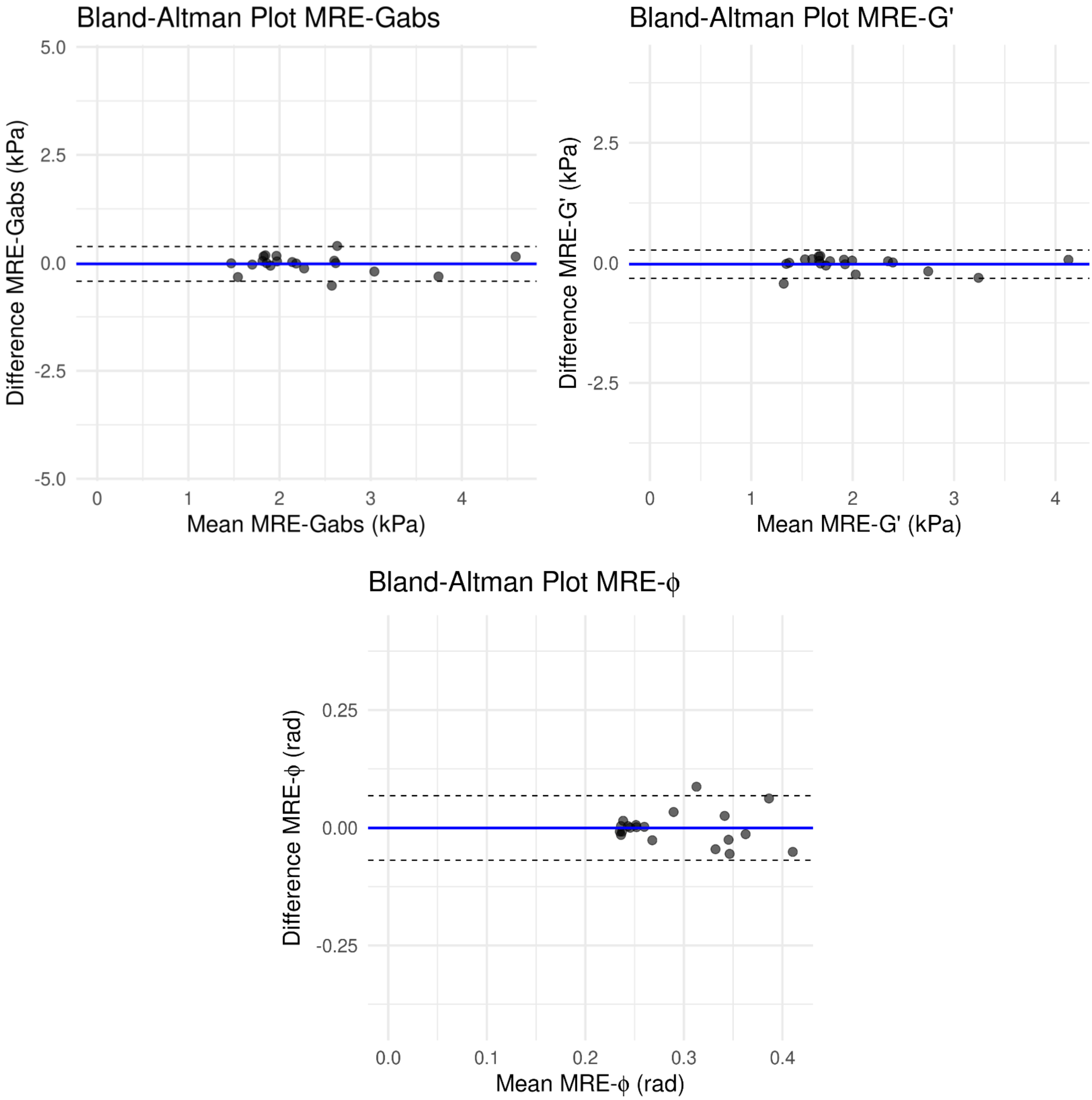

Supplementary Figure S1: Bland-Altman plots for MRE-Gabs, MRE-G’ and MRE-φ, illustrating the agreement between two readers.

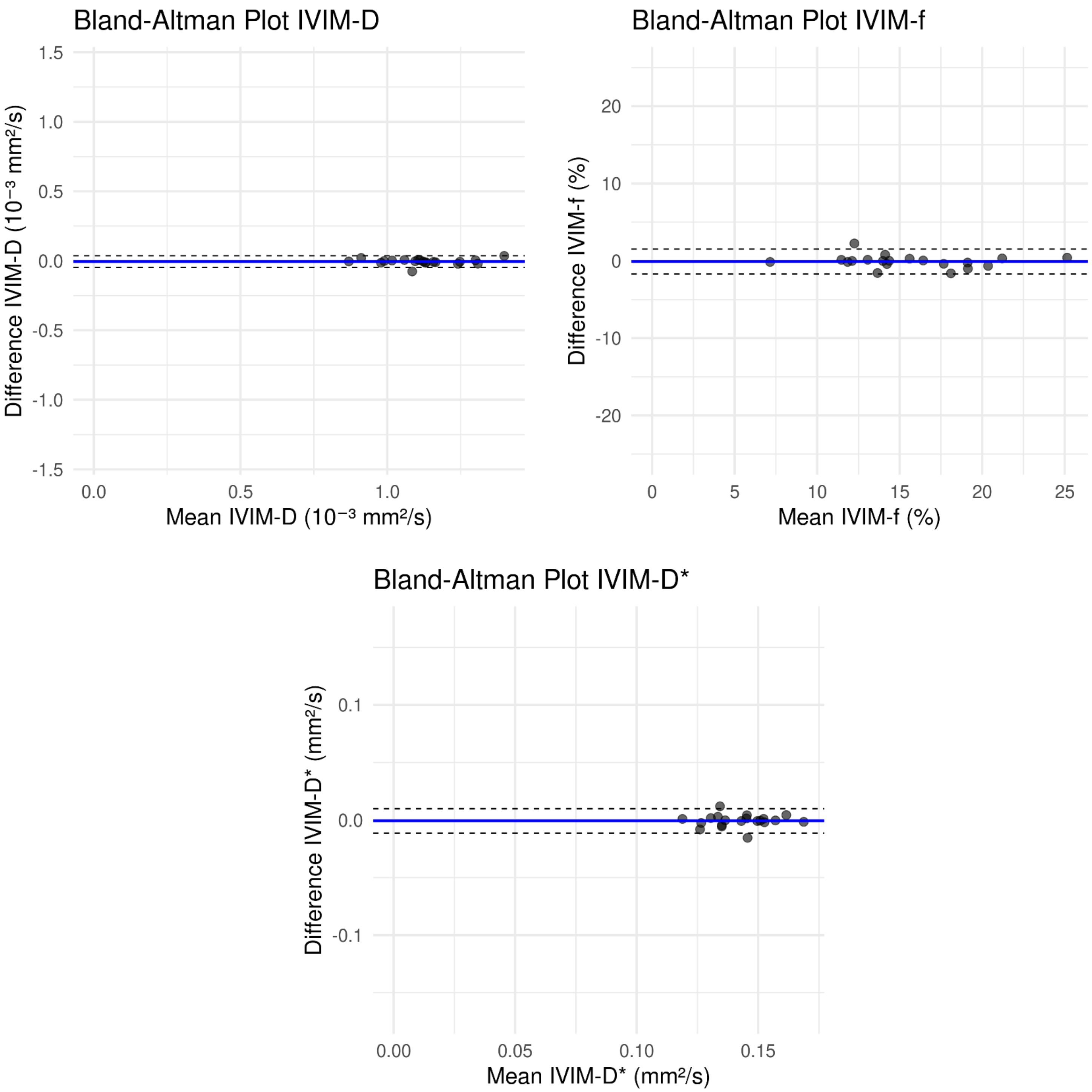

Supplementary Figure S2: Bland-Altman plots for IVIM-D, IVIM-f and IVIM-D*, illustrating the agreement between two readers.

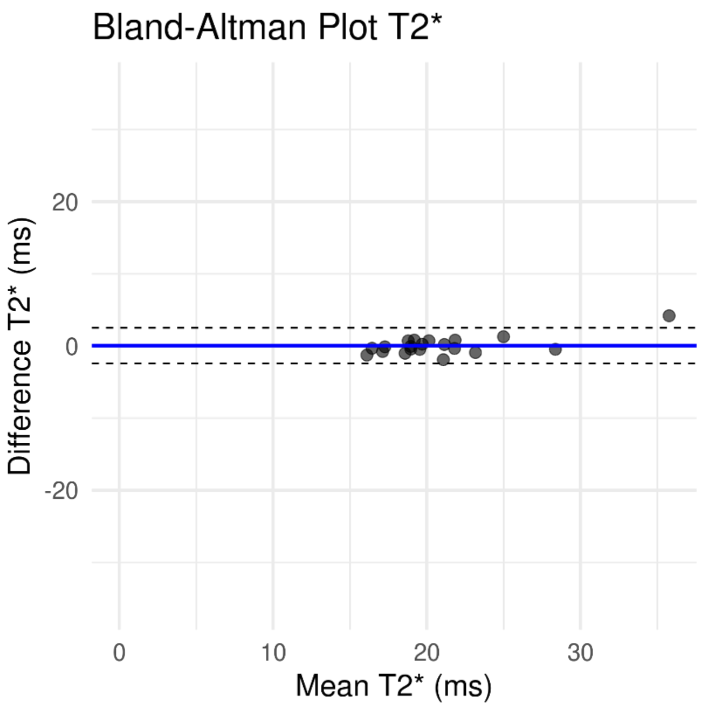

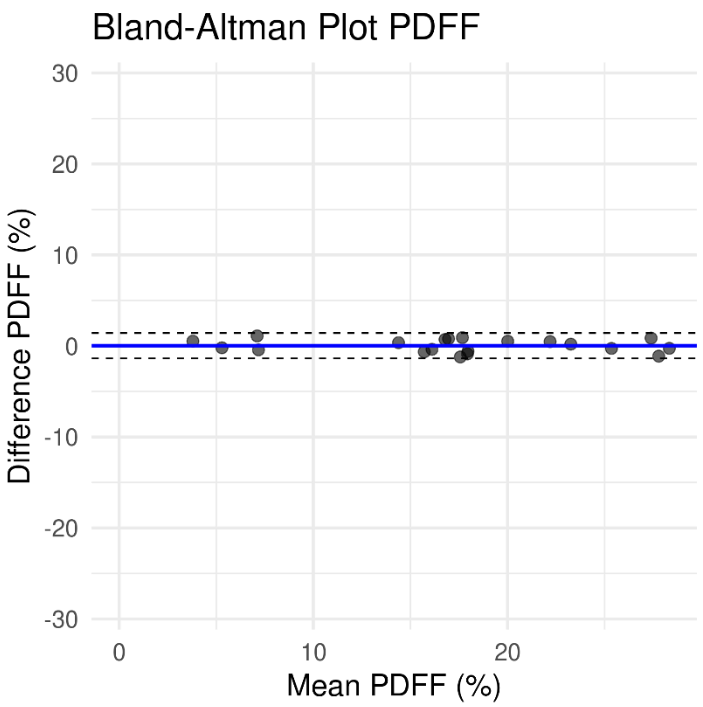

Supplementary Figure S3: Bland-Altman plots for T2* and PDFF, illustrating the agreement between two readers.

3. Supplementary results

Derivation of new composite scores

Univariate logistic regression using 5-fold cross-validation showed that <S3 vs. S3 was best differentiated by cT1 (AUC: 0.84 (±0.06)) and PDFF (AUC: 0.82 (±0.11)) , followed by AST (AUC: 0.72 (±0.15)) (Table S3 and Table S4). VCTE-CAP had an AUC of 0.61 (±0.21). Multivariate logistic regression analysis demonstrated that the combination of cT1 and PDFF with gamma-glutamyl transpeptidase (γGT) had the highest AUC (0.91 (±0.02)): $\log\frac{\mathrm{P}(<\mathrm{S3})}{1-\mathrm{P}(<\mathrm{S3})} = -16.484 + 0.196 * \mathrm{PDFF} + 0.011 * \mathrm{cT1} + 0.011 * \gamma\mathrm{GT}$. Table S5 shows sensitivity, specificity, positive and negative predictive value of the combined score at rule-in and rule-out cut-offs set at 90% sensitivity and 90% specificity, respectively.

The presence of MASH was best detected by cT1 (AUC: 0.76 (±0.06)), followed by AST (0.76 (±0.11)) and MAF5 (AUC: 0.76 (±0.13)) (Table S3 and Table S4). A model combining cT1, AST and waist circumference had the highest AUC (AUC: 0.84 (±0.11)): $\log\frac{\mathrm{P(MASH)}}{1-\mathrm{P(MASH)}} = -11.674 + 0.007 * \mathrm{cT1} + 0.066 * \mathrm{AST} + 0.030 * \mathrm{waist\ circumference}$.

Results using the complete dataset without imputation

Complete data were available for 51 patients, and composite scores were constructed using this subset. The composite scores obtained were as follows:

$$Log(\frac{P(S3)}{1-P(S3)} = -9.955 + 0.135 * PDFF + 0.032 * AST + 0.040 * hip\ circumference$$

$$Log(\frac{P(MASH)}{1-(MASH)} = -31.856 + 0.029 * cT1 + 1.836 * MRE - G' + 0.040 * Age + 2.277 * Sex$$

$$Log(\frac{P(FibroMASH)}{1-P(FibroMASH)} = -33.078 + 0.023 * cT1 + 1.647 * ELF + 0.325 * waist\ circumference$$

$$Log(\frac{P(F2)}{1-P(F2)} = -20.267 + 2.069 * ELF + 0.236 * waist\ circumference - 0.200 * hip\ circumference$$

$$Log(\frac{P(F3)}{1-P(F3)} = 1.485 + 4.374 * MRE - G' - 66.819 * IVIM - f - 0.022 * platelets + 0.041 * waist\ circumference$$

AUC (±SD) of the top five parameters with the highest AUC to differentiate between different histologically-proven stages of MASLD and the composite score using 5-fold cross-validation is shown in Supplementary Table S7.

4. Supplementary tables

	PDFF	MRE	IVIM-DWI	LiverMultiScan® (for cT1 mapping)
Field of view (mm^3)	448 x 320 x 180	448 x 448 x 36	450 x 295 x 188	440 x 330 x 68
Acquisition voxel size (mm^2)	4 x 4	4 x 4	3 x 3	2.3 x 2.3
Slice thickness (mm)	5	4	6	8
Slice gap (mm)	0	0	1	7
Slices	36	9	27	5
Parallel imaging SENSE factor	1.5	3	1.3	2
Repetition time (ms)	150	75	7000	2.42
Echo times (ms)	1.15, 2.33, 3.51, 4.69, 5.86, 7.04	6.91	46	1.05
Flip angle (°)	10	20	90	35
Acquisition duration (s)	18	60	490	60
Respiratory compensation	1 breath-hold	4 breath-holds	Free-breathing	5 breath-holds
Bandwidth (Hz)	1666	2146	20.8 per pixel in phase encoding direction	1173
Fat saturation	-	-	Gradient reversal during slice selection + SPAIR	-
Other	-	Four wave-phase offsets, MEG frequency: 165 Hz, Hadamard encoding, vibrational frequency: 50 Hz.	b-values (s/mm^2): 0, 1, 2, 5, 10, 20, 30, 40, 50, 75, 100, 150, 200, 300, 400, 500, 600, 700. b=0 s/mm^2 consisted of 9 averages, the rest had 3 averages	-

Supplementary table S2: MRI acquisition parameters. Abbreviations: PDFF = proton density fat fraction; MRE = MR elastography; IVIM-DWI = intravoxel incoherent motion diffusion-weighted imaging, cT1 = iron-corrected T1.

	AUC (±SD)				
	<S3 vs. S3	MASH	Fibro-MASH	<F2 vs. ≥F2	<F3 vs. ≥F3
Clinical characteristics					
Age (years)	0.52 (0.18)	0.44 (0.17)	0.60 (0.11)	0.65 (0.11)	0.67 (0.14)
Male sex	0.52 (0.06)	0.45 (0.11)	0.44 (0.19)	0.64 (0.21)	0.44 (0.13)
BMI (kg/m2)	0.60 (0.11)	0.38 (0.10)	0.42 (0.13)	0.60 (0.26)	0.46 (0.06)
T2DM	0.58 (0.11)	0.62 (0.06)	0.64 (0.13)	0.65 (0.21)	0.68 (0.10)
Waist circumference (cm)	0.54 (0.12)	0.58 (0.12)	0.60 (0.21)	0.68 (0.11)	0.57 (0.13)
Hip circumference (cm)	0.58 (0.17)	0.47 (0.09)	0.35 (0.12)	0.56 (0.25)	0.52 (0.08)
Laboratory measurements					
AST (U/L)	0.72 (0.15)	0.76 (0.11)	0.71 (0.13)	0.81 (0.21)	0.59 (0.07)
ALT (U/L)	0.68 (0.14)	0.59 (0.11)	0.56 (0.12)	0.54 (0.29)	0.59 (0.08)
γGT (U/L)	0.63 (0.21)	0.40 (0.23)	0.57 (0.11)	0.42 (0.18)	0.60 (0.16)
Platelets (109)	0.39 (0.07)	0.31 (0.12)	0.39 (0.07)	0.54 (0.24)	0.62 (0.15)
Glucose (mmol/L)	0.54 (0.12)	0.62 (0.19)	0.61 (0.13)	0.62 (0.27)	0.64 (0.13)
HbA1c (mmol/mol)	0.54 (0.11)	0.64 (0.12)	0.65 (0.20)	0.64 (0.06)	0.70 (0.16)
Blood-based biomarkers scores					
FIB4	0.54 (0.16)	0.64 (0.14)	0.65 (0.13)	0.73 (0.20)	0.72 (0.09)
NFS	0.38 (0.08)	0.64 (0.14)	0.66 (0.13)	0.76 (0.21)	0.80 (0.14)
APRI	0.69 (0.15)	0.68 (0.16)	0.68 (0.13)	0.76 (0.09)	0.67 (0.14)
MAF5	0.64 (0.13)	0.76 (0.12)	0.76 (0.13)	0.87 (0.11)	0.73 (0.19)
ELF	0.59 (0.21)	0.70 (0.20)	0.66 (0.17)	0.71 (0.21)	0.76 (0.05)
VCTE parameters					
VCTE-CAP (dB/m)	0.61 (0.21)	0.58 (0.11)	0.56 (0.12)	0.69 (0.11)	0.42 (0.17)
VCTE-LSM (kPa)	0.54 (0.14)	0.63 (0.13)	0.65 (0.16)	0.83 (0.09)	0.78 (0.19)
qMRI parameters					
PDFF (%)	0.82 (0.11)	0.62 (0.16)	0.60 (0.05)	0.52 (0.16)	0.37 (0.11)
T2* (ms)	0.61 (0.12)	0.56 (0.11)	0.42 (0.15)	0.41 (0.06)	0.56 (0.18)
cT1 (ms)	0.84 (0.06)	0.76 (0.06)	0.72 (0.04)	0.63 (0.20)	0.63 (0.18)
MRE-Gabs (kPa)	0.38 (0.13)	0.70 (0.06)	0.70 (0.21)	0.70 (0.17)	0.75 (0.13)

MRE-G' (kPa)	0.57 (0.13)	0.72 (0.10)	0.73 (0.14)	0.76 (0.14)	0.80 (0.10)
MRE-φ (rad)	0.62 (0.12)	0.51 (0.10)	0.36 (0.24)	0.53 (0.15)	0.59 (0.11)
IVIM-D (10^{-3} mm^2/s)	0.64 (0.24)	0.62 (0.20)	0.67 (0.12)	0.68 (0.19)	0.66 (0.20)
IVIM-f (%)	0.39 (0.09)	0.69 (0.10)	0.66 (0.13)	0.68 (0.11)	0.71 (0.15)
IVIM-D* (mm^2/s)	0.67 (0.13)	0.43 (0.20)	0.56 (0.10)	0.57 (0.22)	0.62 (0.08)

Supplementary table S3: AUC (±SD) of clinical characteristics, laboratory measurements, blood-based biomarkers scores, VCTE parameters and qMRI parameters in differentiation between histologically-proven stages of MASLD (<S3 vs. ≥S3; MASH; fibro-MASH; <F2 vs. ≥F2; <F3 vs. ≥F3) and the combined score using 5-fold cross-validation.

AUC = area under the curve; SD = standard deviation; S3 = advanced steatosis; MASH = metabolic dysfunction-associated steatohepatitis; F2 = significant fibrosis; F3 = advanced fibrosis; BMI = body mass index; T2DM = type 2 diabetes mellitus; AST = aspartate transferase; ALT = alanine transferase; γGT = gamma-glutamyltransferase; HbA1c = glycated hemoglobulin; FIB4 = fibrosis-4 score; NFS = non-alcoholic fatty liver fibrosis score; APRI = AST to platelet ratio index; MAF5 = metabolic dysfunction-associated fibrosis-5 score; ELF = enhanced liver fibrosis-score; VCTE = vibration controlled transient elastography; CAP = controlled attenuation parameter; LSM = liver stiffness measurement; PDFF = proton-density fat fraction; cT1 = iron-corrected T1; MRE = MR elastography, Gabs = stiffness; G' = elasticity; φ = phase angle; IVIM = intravoxel incoherent motion; D = diffusion coefficient; f = perfusion fraction; D* = pseudo-diffusion coefficient.

Stage of MASLD	Parameter	AUC (±SD)
<S3 vs. S3	cT1	0.84 (0.06)
	PDFF	0.82 (0.11)
	AST	0.72 (0.15)
	APRI	0.69 (0.15)
	ALT	0.68 (0.14)
	Composite score	0.91 (0.02)
MASH	cT1	0.76 (0.06)
	AST	0.76 (0.11)
	MAF5	0.76 (0.12)
	MRE-G’	0.72 (0.10)
	MRE-Gabs	0.70 (0.06)
	Composite score	0.84 (0.11)

Supplementary table S4: AUC (±SD) of the top five parameters with the highest AUC to differentiate between different histologically-proven stages of MASLD (<S3 vs. S3; MASH) and the composite score using 5-fold cross-validation.

	<S3 vs. S3	MASH	Fibro-MASH	<F2 vs. ≥F2	<F3 vs. ≥F3
Rule-in cut-off					
Threshold	-1.77	-0.27	-0.68	0.61	-1.60
Sensitivity	0.90	0.90	0.90	0.90	0.90
Specificity	0.72	0.42	0.37	0.38	0.42
PPV	0.55	0.79	0.76	0.89	0.51
NPV	0.96	0.65	0.63	0.42	0.88
Rule-out cut-off					
Threshold	-0.56	1.18	0.87	2.16	-0.03
Sensitivity	0.71	0.60	0.49	0.67	0.66
Specificity	0.90	0.90	0.90	0.90	0.90
PPV	0.74	0.90	0.91	0.98	0.82
NPV	0.89	0.47	0.44	0.32	0.80

Supplementary table S5: Diagnostic performance of the combined scores at rule-in and rule-out cut-offs set at 90% sensitivity and 90% specificity, respectively.

S3 = advanced steatosis; MASH = metabolic dysfunction-associated steatohepatitis; F2 = significant fibrosis; F3 = advanced fibrosis; PPV = positive predictive value; NPV = negative predictive value.

**Stage of MASLD**	**Parameter**	**AUC (95% CI)**	**p-value (compared to composite score)**
<S3 vs. S3	Composite score	0.91 (0.84-0.97)	-
	cT1	0.83 (0.73-0.93)	**0.039**
	PDFF	0.82 (0.73-0.92)	**<0.001**
	ALT	0.70 (0.58-0.83)	**<0.001**
	VCTE-CAP	0.59 (0.47-0.71)	**<0.001**
MASH	Composite score	0.82 (0.73-0.92)	-
	MAF5	0.76 (0.66-0.87)	0.271
	cT1	0.76 (0.65-0.87)	0.091
	AST	0.74 (0.63-0.85)	0.080
	MR-MASH	0.62 (0.49-0.76)	**<0.001**

Supplementary table S6: AUC (95% CI) of the composite scores and other non-invasive tests for the differentiation between different histologically-proven stages of MASLD (<S3 vs. S3; MASH) and comparison of AUC of the composite score with established non-invasive tests using DeLong test.

S3 = advanced steatosis; MASH = metabolic dysfunction-associated steatohepatitis; AUC = area under the curve; CI=confidence interval; AST = aspartate transferase; ALT = alanine transferase; MAF5 = metabolic dysfunction-associated fibrosis-5 score; VCTE = vibration controlled transient elastography; CAP = controlled attenuation parameter; PDFF = proton-density fat fraction; cT1 = iron-corrected T1.

Stage of MASLD	Parameter	AUC (±SD)
<S3 vs. ≥S3	cT1	0.81 (0.11)
	PDFF	0.78 (0.17)
	ALT	0.77 (0.14)
	IVIM-D	0.72 (0.15)
	AST	0.71 (0.18)
	Composite score	0.87 (0.13)
MASH	cT1	0.87 (0.11)
	AST	0.81 (0.13)
	MAF5	0.81 (0.16)
	MRE-G'	0.75 (0.30)
	HbA1c	0.75 (0.25)
	Composite score	0.95 (0.06)
Fibro-MASH	cT1	0.80 (0.12)
	MAF5	0.78 (0.17)
	ELF	0.75 (0.22)
	MRE-G'	0.75 (0.11)
	APRI	0.75 (0.08)
	Composite score	0.91 (0.09)
<F2 vs. ≥F2	VCTE-LSM	0.89 (0.22)
	MAF5	0.84 (0.09)
	ELF	0.80 (0.15)
	FIB4	0.79 (0.06)
	NFS	0.79 (0.26)
	Composite score	0.94 (0.09)
<F3 vs. ≥F3	MRE-G'	0.90 (0.12)
	VCTE-LSM	0.86 (0.15)
	ELF	0.85 (0.15)
	MRE-Gabs	0.84 (0.15)
	FIB4	0.82 (0.09)
	Composite score	0.96 (0.06)

Supplementary table S7: AUC (±SD) of the top five parameters with the highest AUC to differentiate between different histologically-proven stages of MASLD and the composite score using 5-fold cross-validation in those participants only with complete data (n=51).

5. Supplementary figures

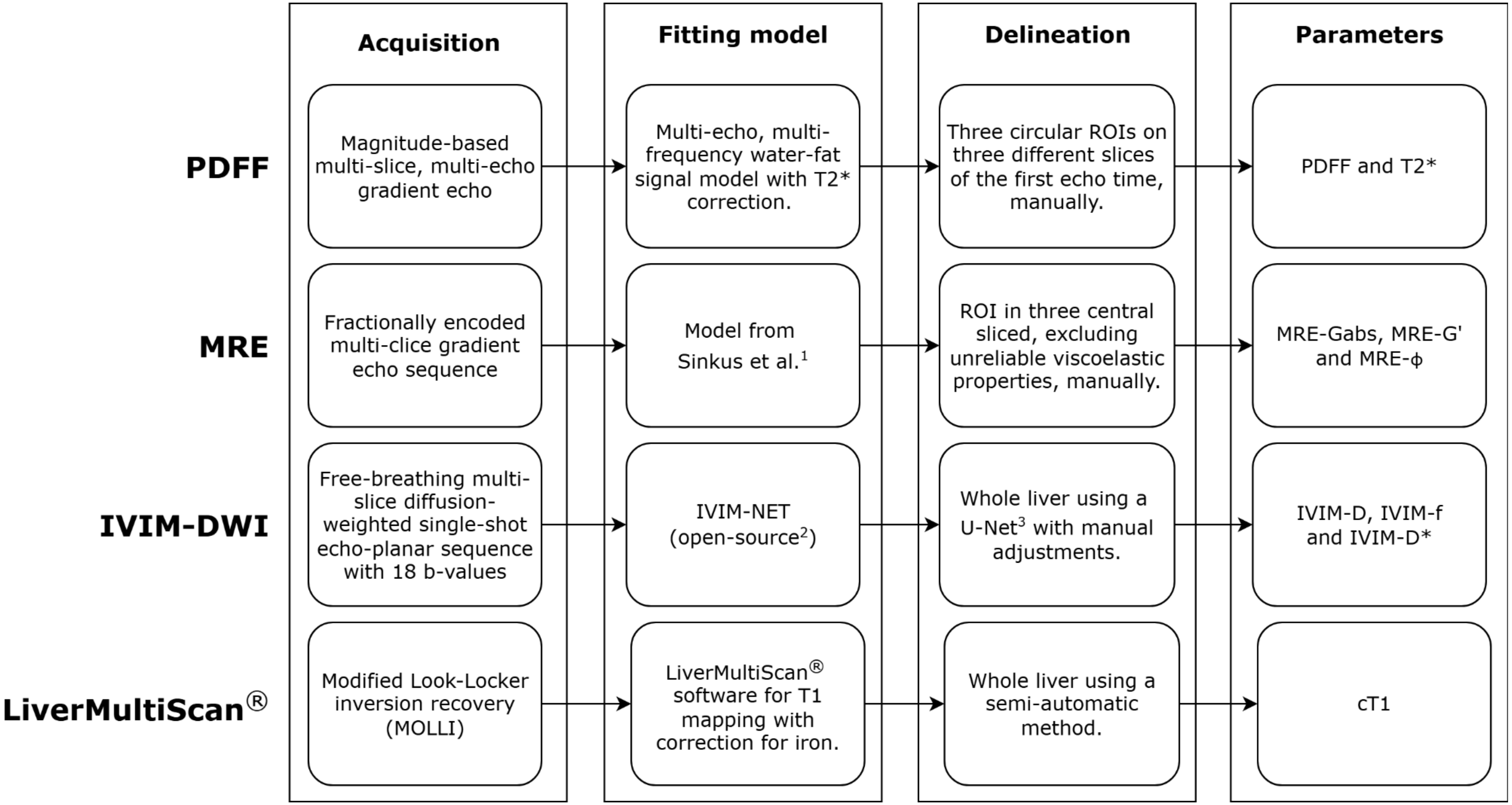

Supplementary figure 4: Flowchart showing the acquisition settings and post-processing steps of all quantitative MRI scans performed in this study.

[1] Sinkus R, Lambert S, Abd-Elmoniem KZ et al (2018) Rheological determinants for simultaneous staging of hepatic fibrosis and inflammation in patients with chronic liver disease. NMR Biomed 31:e3956

[2] https://github.com/oliverchampion/IVIMNET

[3] https://github.com/dilaratank/BScThesis-AutoLiverSeg

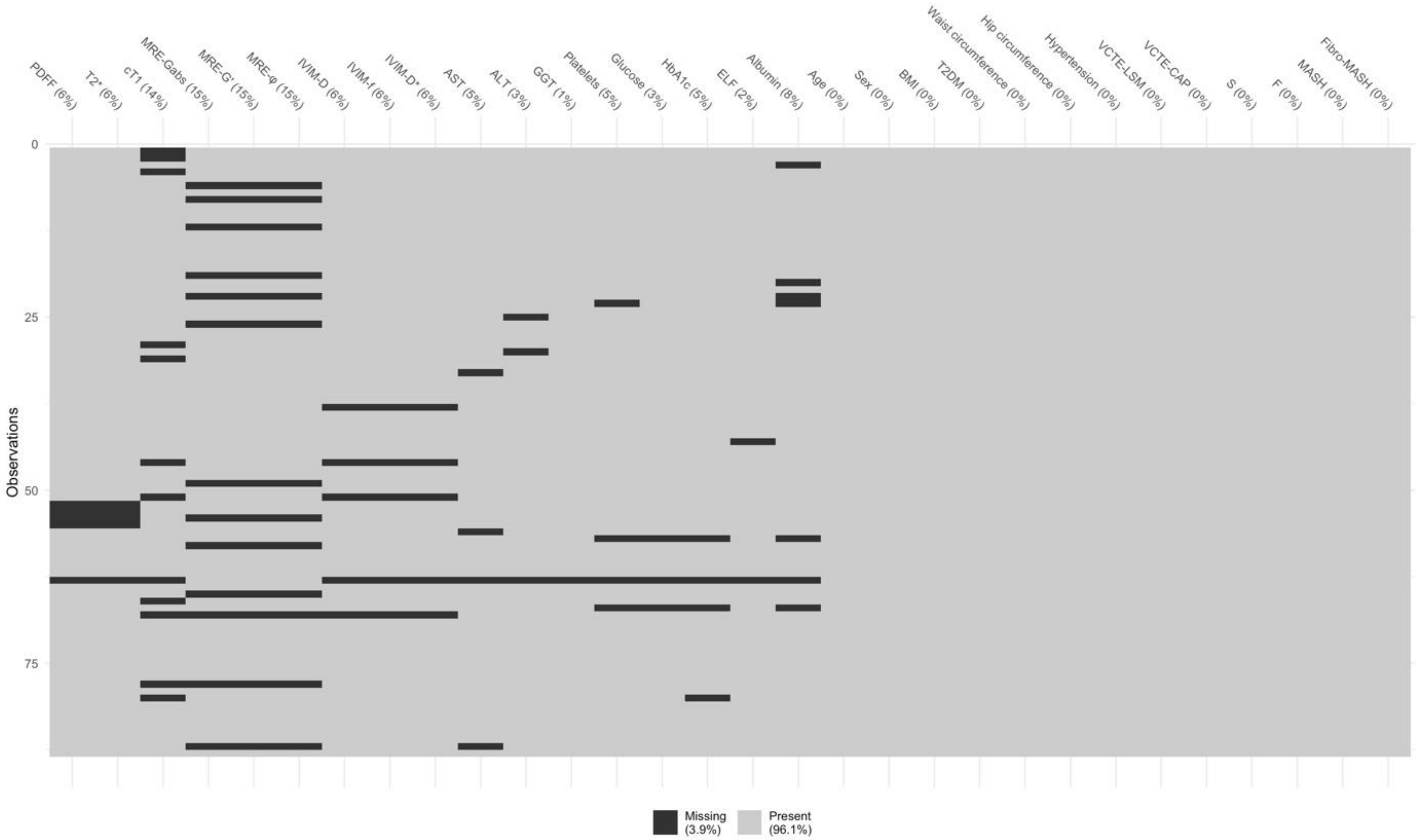

Supplementary figure 5: Plot of missing data per variable.

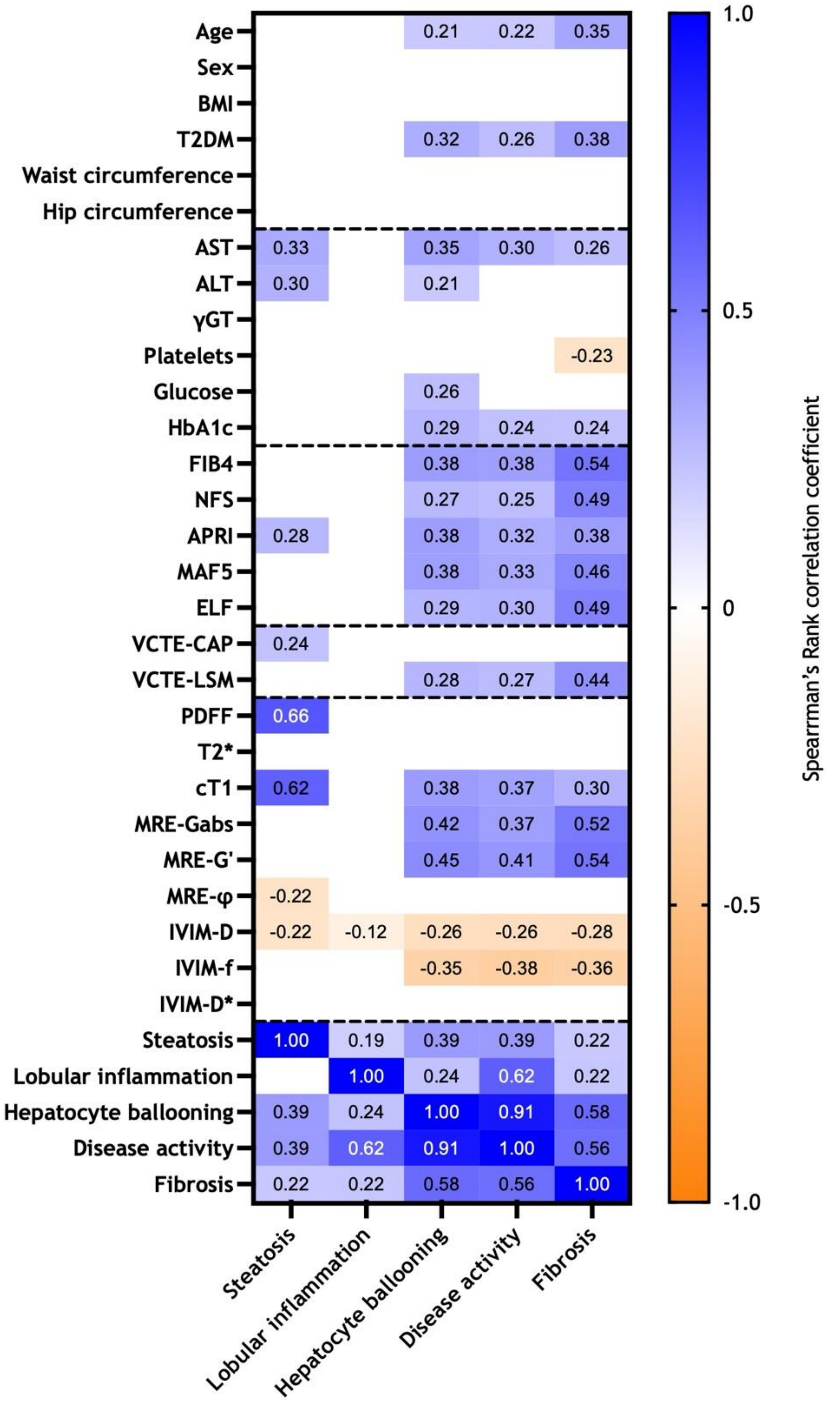

Supplementary figure 6: Correlogram showing the significant Spearman's R correlation coefficients between the qMRI parameters, VCTE parameters, laboratory measurements, blood-based biomarker scores, clinical characteristics and liver histology (rows), and liver histology (columns). Non-significant correlations are left blank.

Abbreviations: VCTE = vibration controlled transient elastography; BMI = body mass index; T2DM = type 2 diabetes mellitus; AST = aspartate transferase; ALT = alanine transferase; γGT = gamma-glutamyltransferase; FIB4 = fibrosis-4 score; NFS = NAFLD fibrosis score; APRI = AST to platelet ratio index; MAF5 = metabolic dysfunction-associated fibrosis-5 score; ELF = enhanced liver fibrosis-score; VCTE = vibration-controlled transient elastography; CAP = controlled attenuation parameter; LSM = liver stiffness measurement; PDFF = proton-density fat fraction; cT1 = iron-corrected T1; MRE = magnetic resonance elastography; Gabs = stiffness; G' = elasticity; φ = phase angle; D = diffusion coefficient; f = perfusion fraction; D* = pseudo-diffusion coefficient.

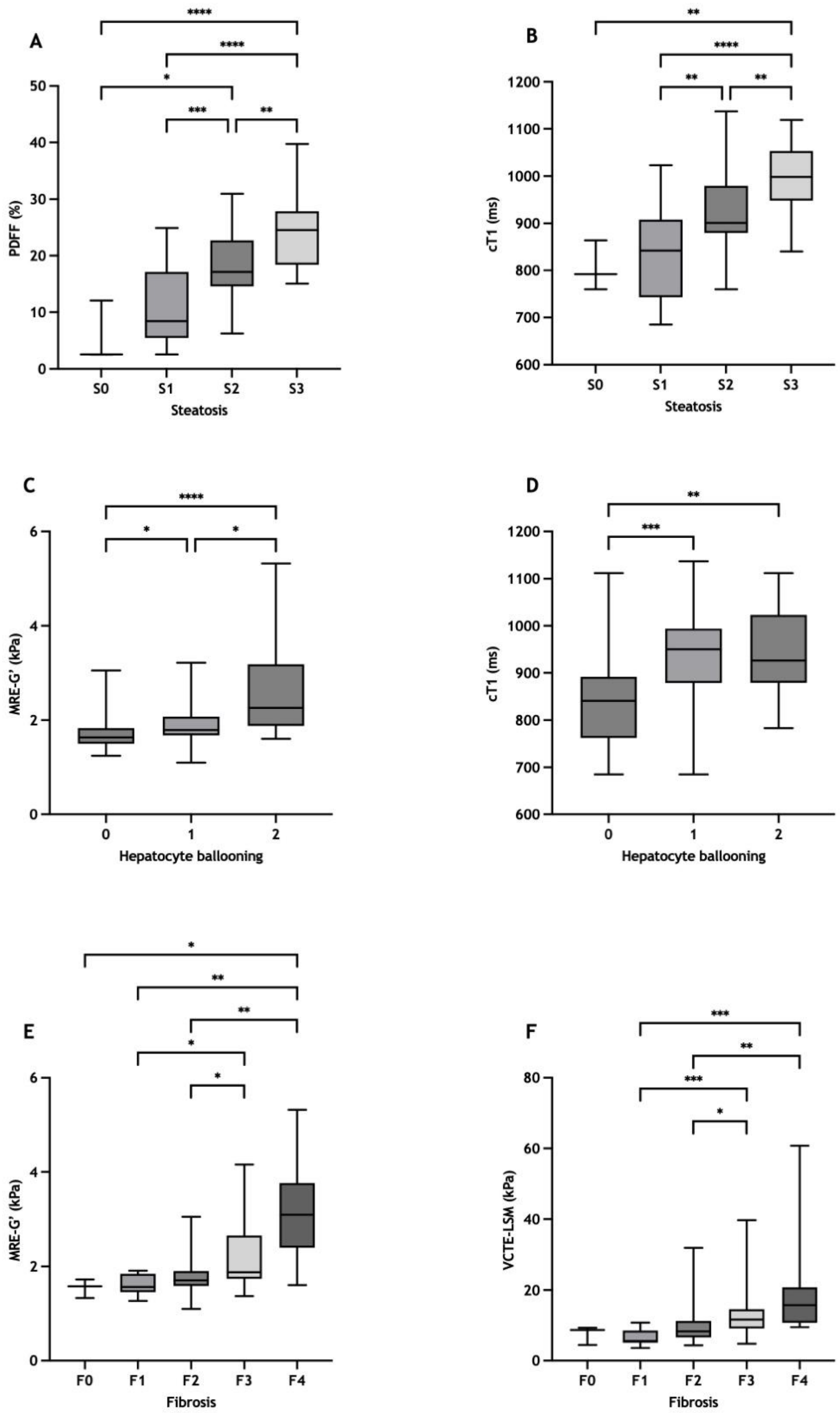

Supplementary figure 7: Boxplots of the two highest Pearson correlation coefficient per histological grade. PDFF and cT1 versus histological steatosis grades **(A, B)**, MRE-G' and cT1 versus hepatocyte ballooning scores **(C, D)**, and MRE-G' and VCTE-LSM versus histological fibrosis grades **(E, F)**.
* = p<0.05; ** = p<0.01; *** = p<0.001; **** = p<0.0001.

Abbreviations: PDFF = proton-density fat fraction; cT1 = iron-corrected T1; MRE = magnetic resonance elastography; MRE-G' = MR elastography derived elasticity; VCTE-LSM = vibration-controlled transient elastography derived liver stiffness measurement.

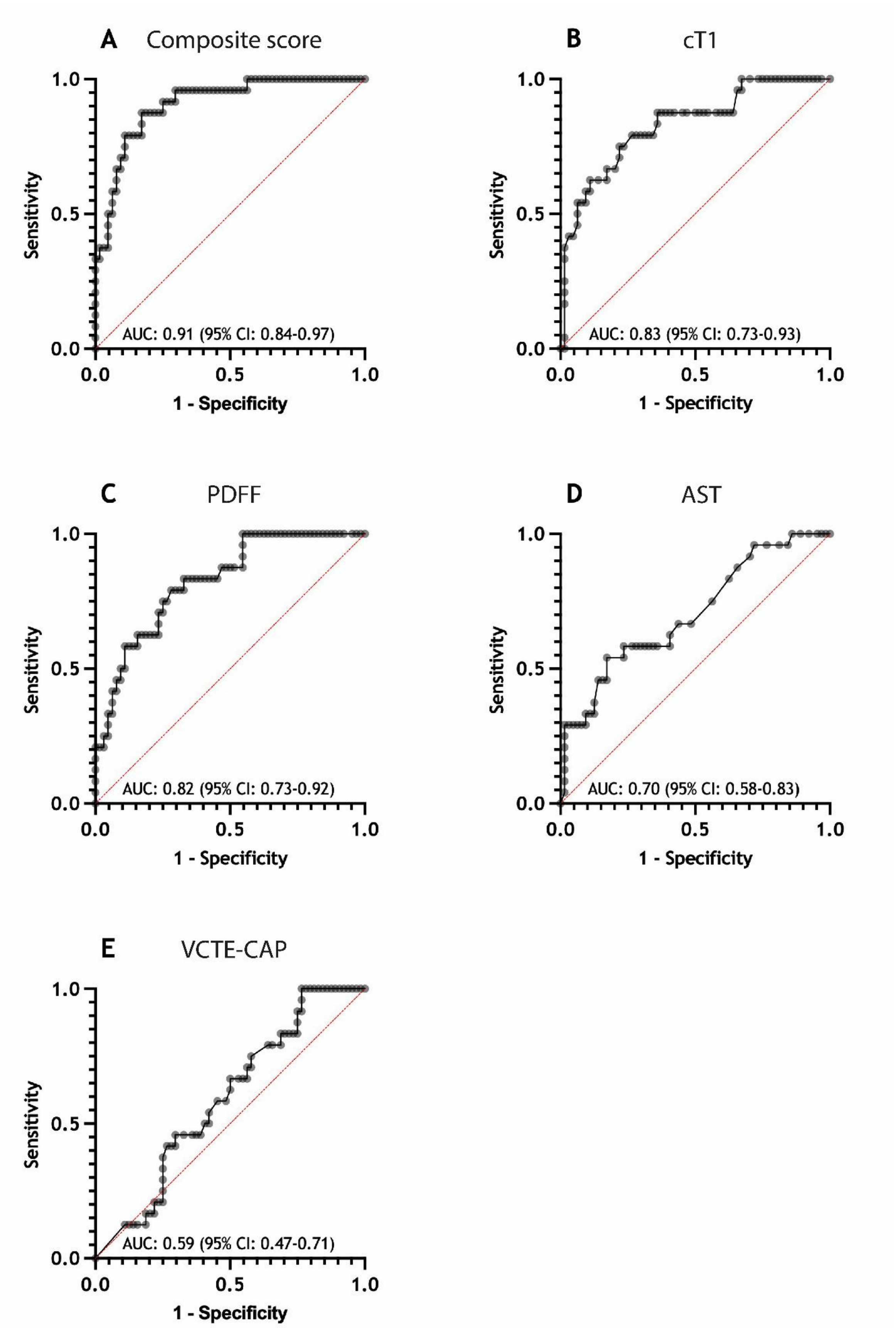

Supplementary figure 8: ROC curves for the composite scores and other non-invasive tests for the differentiation of <S3 vs. S3.

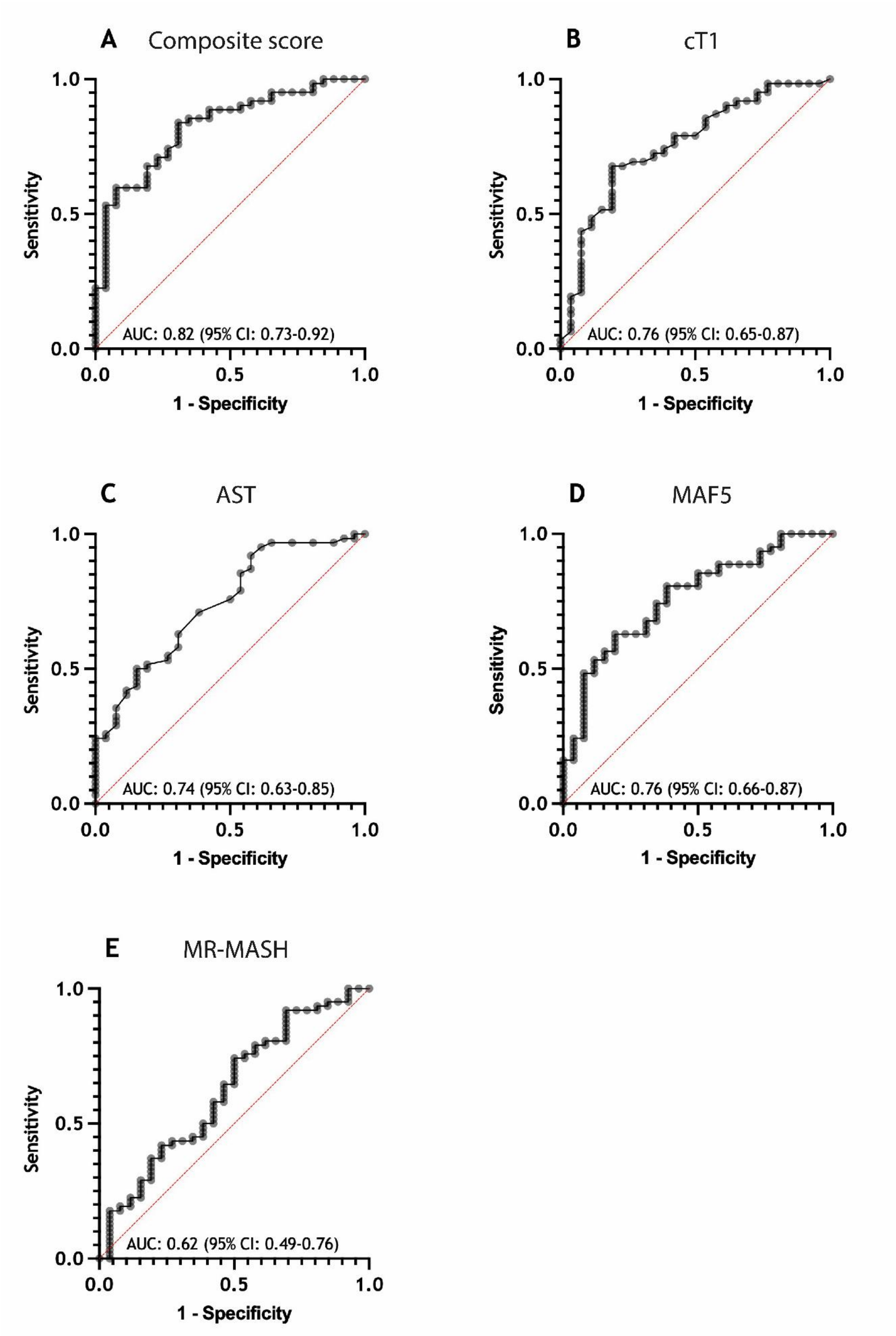

Supplementary figure 9: ROC curves for the composite scores and other non-invasive tests for the differentiation of MASH.

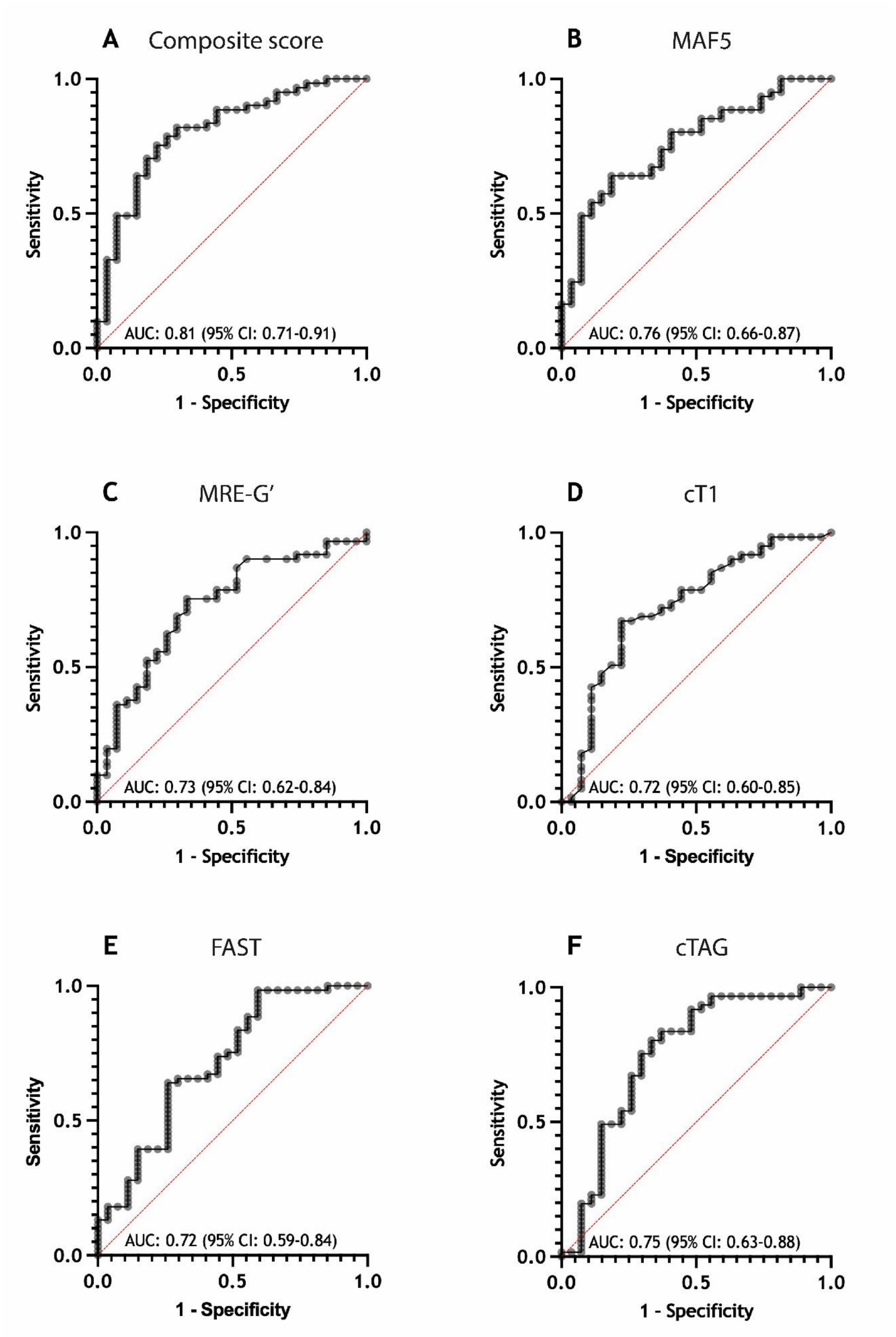

Supplementary figure 10: ROC curves for the composite scores and other non-invasive tests for the differentiation of fibro-MASH.

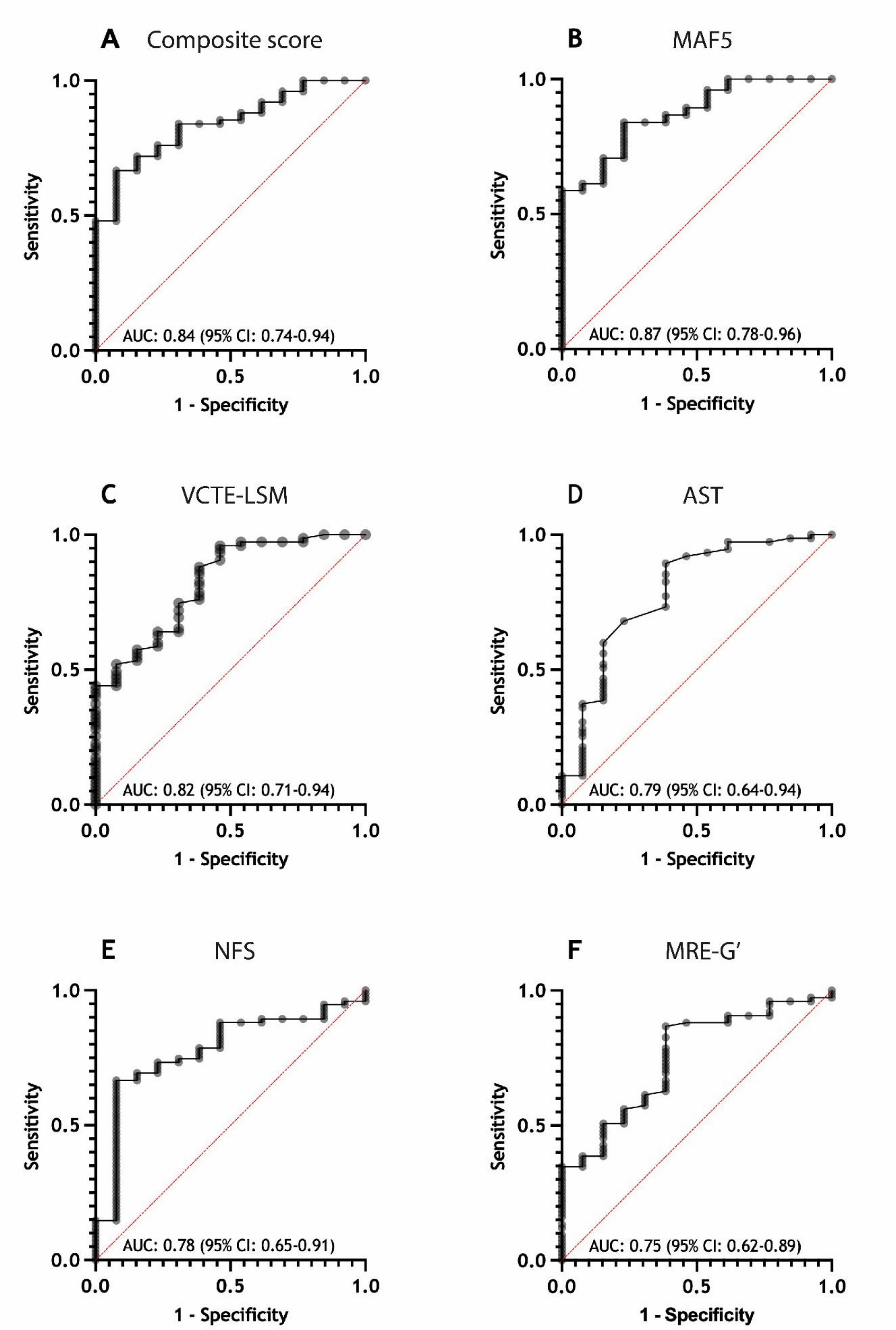

Supplementary figure 11: ROC curves for the composite scores and other non-invasive tests for the differentiation of <F2 vs. ≥F2.

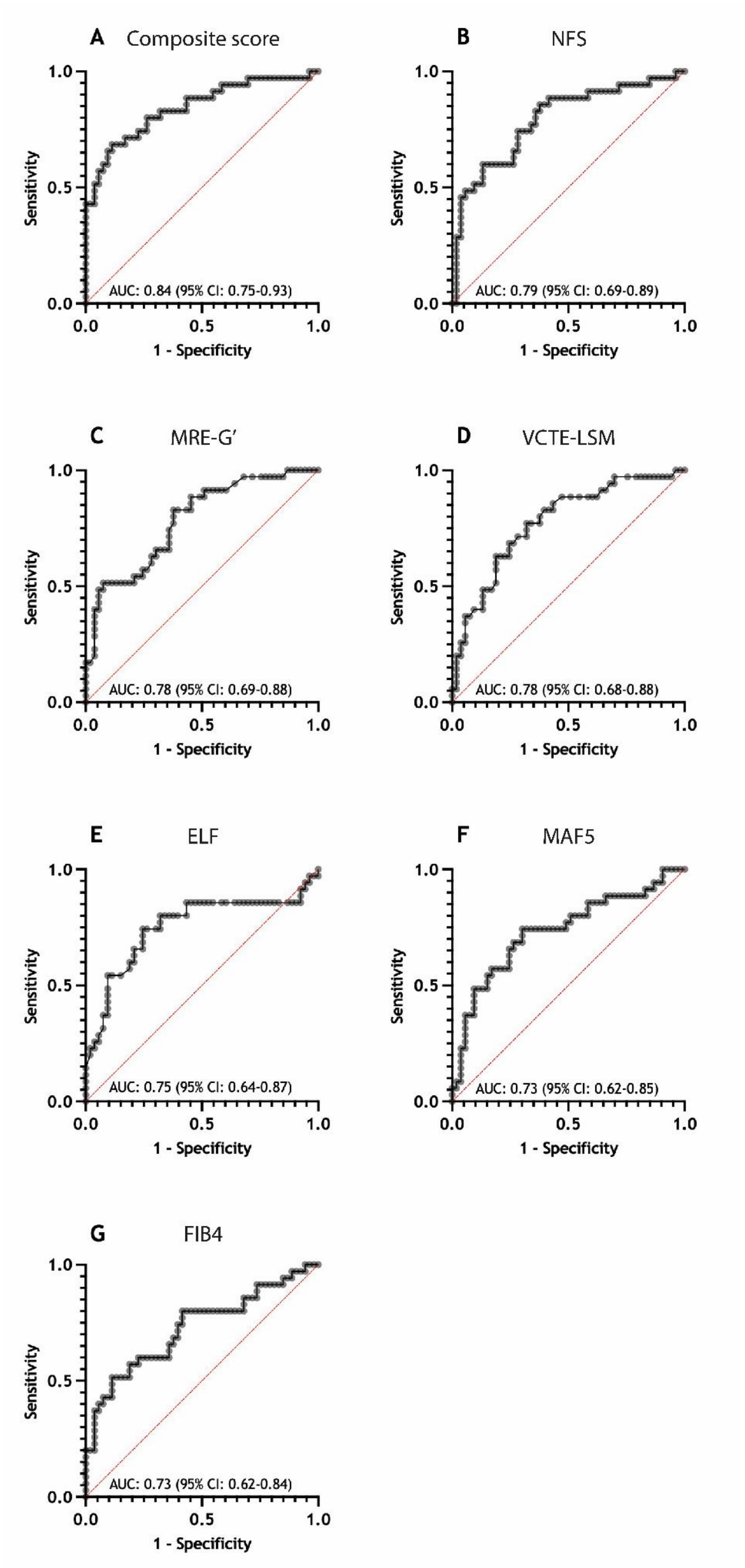

Supplementary figure 12: ROC curves for the composite scores and other non-invasive tests for the differentiation of <F3 vs. ≥F3.
